# Erythrocyte nano-ghosts with dual optical and magnetic resonance characteristics

**DOI:** 10.1117/1.JBO.29.8.085001

**Published:** 2024-08-20

**Authors:** Chi-Hua Lee, Shamima Zaman, Vikas Kundra, Bahman Anvari

**Affiliations:** a University of California, Riverside, Department of Biochemistry, Riverside, California, United States; b University of California, Riverside, Department of Bioengineering, Riverside, California, United States; c University of Maryland School of Medicine, Department of Diagnostic Radiology and Nuclear Medicine, Baltimore, Maryland, United States; d University of Maryland, Stuart and Marlene Greenbaum Comprehensive Cancer Center, Baltimore, Maryland, United States

**Keywords:** biomimetics, fluorescence, halogenated dyes, nanoparticles, near-infrared, red blood cells, spectroscopy

## Abstract

**Significance:**

Fluorescent organic dyes provide imaging capabilities at cellular and sub-cellular levels. However, a common problem associated with some of the existing dyes such as the US FDA–approved indocyanine green (ICG) is their weak fluorescence emission. Alternative dyes with greater emission characteristics would be useful in various imaging applications. Complementing optical imaging, magnetic resonance (MR) imaging enables deep tissue imaging. Nano-sized delivery systems containing dyes with greater fluorescence emission as well as MR contrast agents present a promising dual-mode platform with high optical sensitivity and deep tissue imaging for image-guided surgical applications.

**Aim:**

We have engineered a nano-sized platform, derived from erythrocyte ghosts (EGs), with dual near-infrared fluorescence and MR characteristics by co-encapsulation of a brominated carbocyanine dye and gadobenate dimeglumine (Gd-BOPTA).

**Approach:**

We have investigated the use of three brominated carbocyanine dyes (referred to as BrCy106, BrCy111, and BrCy112) with various degrees of bromination, structural symmetry, and acidic modifications for encapsulation by nano-sized EGs (nEGs) and compared their resulting optical characteristics with nEGs containing ICG.

**Results:**

We find that asymmetric dyes (BrCy106 and BrCy112) with one dibromobenzene ring offer greater fluorescence emission characteristics. For example, the relative fluorescence quantum yield (ϕ) for nEGs fabricated using 100  μM of BrCy112 is ∼41-fold higher than nEGs fabricated using the same concentrations of ICG. The dual-mode nEGs containing BrCy112 and Gd-BOPTA show a nearly twofold increase in their ϕ as compared with their single optical mode counterpart. Cytotoxicity is not observed upon incubation of SKOV3 cells with nEGs containing BrCy112.

**Conclusions:**

Erythrocyte nano-ghosts with dual optical and MR characteristics may ultimately prove useful in various biomedical imaging applications such as image-guided tumor surgery where MR imaging can be used for tumor staging and mapping, and fluorescence imaging can help visualize small tumor nodules for resection.

## 1 Introduction

**Fig. 1:**
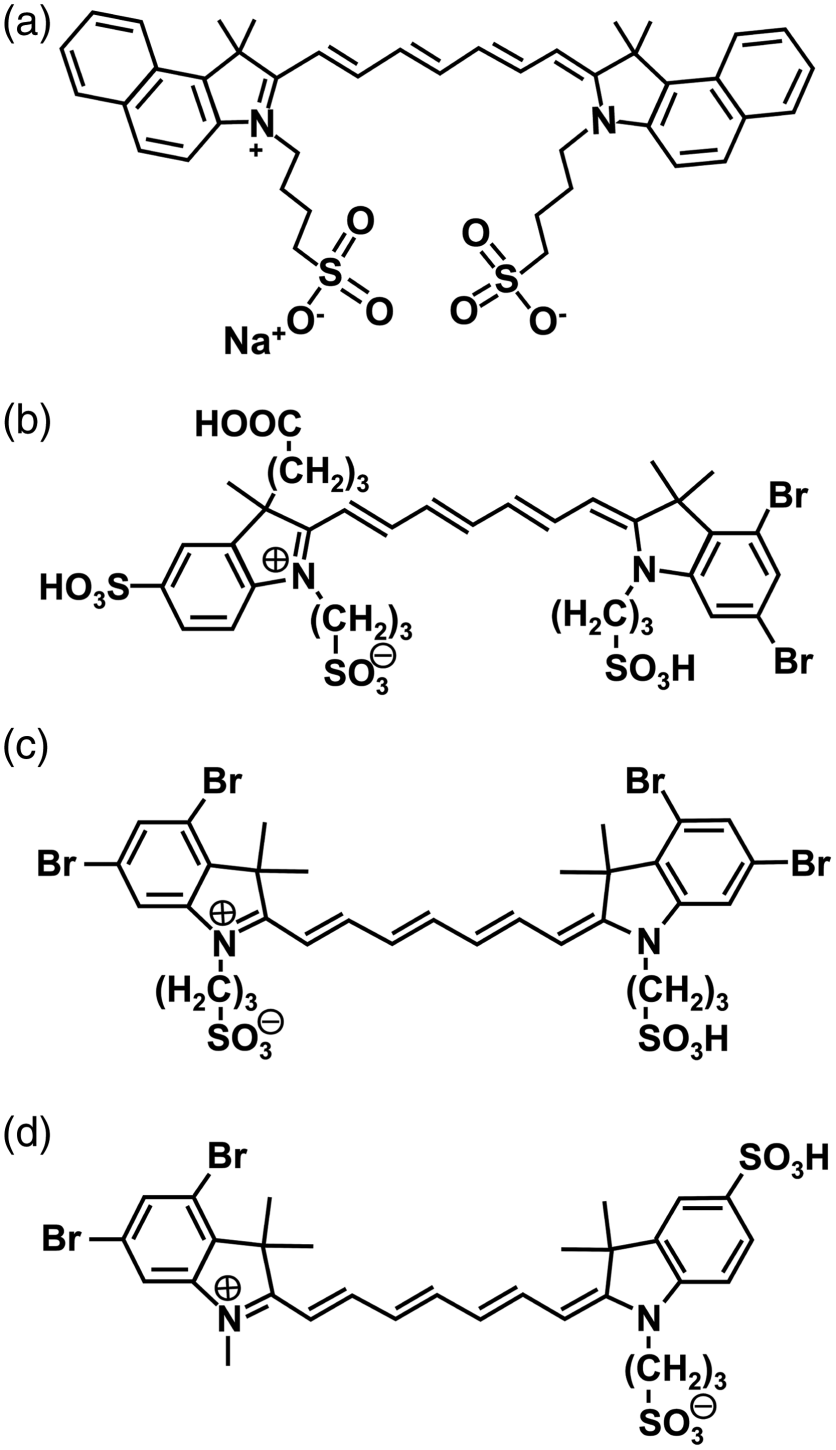
Molecular structures of (a) ICG, (b) BrCy106, (c) BrCy111, and (d) BrCy112.

Fluorescent organic dyes provide an enabling platform for image-guided surgery and other clinical applications.^1^^–^^3^ In particular, when activated by near-infrared (NIR) light (700 to 2500 nm), such materials mediate fluorescence imaging on the order of ∼1  cm (depending on wavelength and tissue type) and enhance the image contrast as a result of the lower autofluorescence in the NIR spectral band.^1^^,^^4^^–^^6^ The US FDA–approved indocyanine green (ICG) [molecular weight (MW) = 775 Da] remains the principal NIR dye used in specific clinical applications such as ophthalmic angiography^7^ and liver function assessment^8^ and has been investigated for its utility in intraoperative cancer imaging.^9^^–^^13^ It is a dye with two tricarbocyanine systems connected by a polyene bridge and terminal sulfonate groups attached to each of the nitrogen-containing heterocycles [Fig. 1(a)]. The delocalization of the electrons across the bridge gives rise to NIR absorption and fluorescence properties of ICG and other carbocyanine dyes.^14^ Despite its long clinical usage dating back to 1950,^15^ drawbacks of ICG include weak fluorescence emission with relative fluorescence quantum yields of ∼2% and 4% in water and albumin solution, respectively, at low concentrations (∼1  μM),^16^ and rapid clearance from the bloodstream in a biexponential manner with a half-life of less than 10 min.^7^^,^^17^^,^^18^ Previous studies indicated that once within the vasculature, ICG binds to albumin and lipoproteins and is transported to the liver where it is then excreted by the hepatobiliary mechanism.^19^^–^^21^

**Fig. 2:**
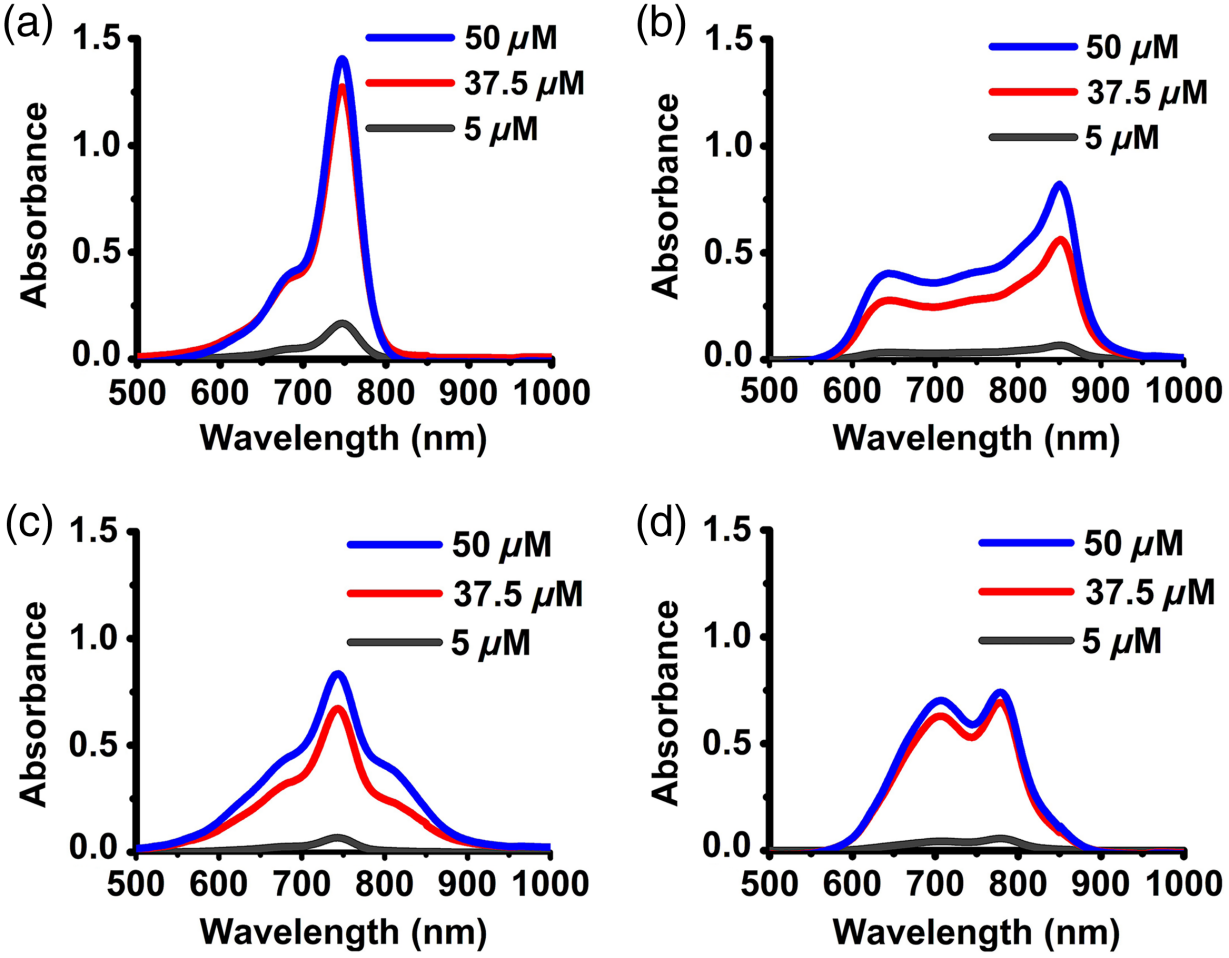
Concentration-dependent absorption spectra of (a) BrCy106, (b) BrCy111, (c) BrCy112, and (d) ICG in 1X PBS.

To address the weak emission of ICG, new NIR fluorophores have been under development and investigation.^5^^,^^22^^–^^28^ One class of organic fluorophores is carbocyanine dyes with added heavy halogen elements such as bromine, chlorine, fluorine, or iodine.^29^^–^^32^ In particular, specific brominated carbocyanine dyes containing indoline and indolenine groups are the subject of this study: (1) BrCy106 (MW = 934.73 Da), an unsymmetric dye with one dibromobenzene ring, carboxyl, and sulfonic acid attached to the indolenine group and further modified with the addition of sulfonic acid and sulfonate (conjugate base of sulfonic acid) to the indoline and indolenine groups, respectively [Fig. 1(b)]; (2) BrCy111 (MW = 940.40 Da), a more symmetric dye with two dibromobenzene rings and sulfonic acid and sulfonate attached to the indoline and indolenine groups, respectively [Fig. 1(c)]; and (3) BrCy112 (MW = 754.55 Da), another unsymmetric dye with one dibromobenzene ring and sulfonic acid and sulfonate attached to the indoline group [Fig. 1(d)].

In a previous study, we determined that the peak emission intensity of the BrCy106 dye containing a N-hydroxysuccinimide (NHS)-activated carboxyl group emitted at 780 nm (when photoexcited at 720 nm) was ∼65 times higher than that of ICG emitted at 775 nm (in response to 680-nm excitation) when dissolved in an aqueous buffer containing NaAc and MgAc, at the same concentration of 20  μg/ml.^33^ The superior emission characteristics of the brominated carbocyanine dye, attributed to the presence of the bromine elements that reduce the non-radiative relaxation pathways involving C–H vibrations modes, motivate further investigations of such dyes presented in this study. We had also previously encapsulated BrCy106–NHS by capsid proteins obtained from the plant-infecting brome mosaic virus and used the resulting nano-constructs for NIR fluorescence imaging of intraperitoneal ovarian tumors in mice.^33^^,^^34^ Encouraged by these results, herein, we expand our studies to other brominated carbocyanine dyes shown in Fig. 1.

To shield ICG from binding to plasma proteins and extend its circulation, it has been encapsulated into various nano-sized constructs, including those composed of micelles, liposomes, polymers, and viral capsid proteins.^35^^–^^45^ We reported the first demonstration of encapsulating ICG within nano-constructs derived from erythrocytes [red blood cells (RBCs)]^46^ and have shown their utility in mediating NIR fluorescence imaging and photo-destruction of tumors in mice.^47^^–^^49^ The use of RBCs as delivery systems has also been reported by other investigators.^50^^–^^53^

A particular feature of RBCs is their naturally long circulation time (∼90 to 120 days), attributed to the presence of specific membrane proteins, including CD47, which impedes phagocytosis by macrophages; CD55, which protects against cell lysis by the complement system; and CD59, which blocks the assembly of membrane–attack complexes.^54^^–^^57^ Our proteomics analysis, based on tandem mass spectroscopy, indicates that CD47, CD55, and CD59 are retained on the surface of ICG-containing nano-constructs fabricated from RBCs.^58^ This built-in immune-inhabiting machinery, absent on synthetic nano-constructs such as liposomes but available on RBC nano-ghosts, is a key distinguishing characteristic needed for prolonged circulation of the particles. For example, it has been reported that the circulation half-life of ICG-containing liposomes with a mean diameter of ∼200  nm was only ∼10  min in immunodeficient Balb/C mice incapable of producing T cells.^59^ In a previous study, we found that the half-life of RBC nano-ghosts containing ICG in the bloodstream of healthy Swiss Webster (hSW) mice was nearly 2 h, and ∼11% of these constructs were still within the vasculature 48 h post-tail vein injection.^60^ Another advantage of RBC nano-ghosts is that as materials fabricated autologously, they present personalized constructs and are expected to be highly biocompatible, non-immunogenic, and non-toxic. We have shown that the histological sections of various organs of hSW mice injected with RBC nano-ghosts, as well as the hematological panels and serum biochemistry assaying for liver and kidney functions, were similar to those from mice injected with phosphate-buffered saline (PBS) control.^60^ Our previous acute immunogenic studies based on measurements of specific cytokines following intravascular administration of RBC nano-ghosts in hSW mice have shown that the mean values of interleukin (IL)-6, IL-10, and monocyte chemoattractant protein-1 in the blood serum at up to 6 h post-injection were not significantly different than those in response to PBS administration.^61^

Herein, for the first time, we report on the encapsulation of three brominated carbocyanine fluorophores (BrCy106, BryCy111, and BrCy112) into RBC nano-ghosts and compare the optical characteristics of these particles with ICG-containing RBC nano-ghosts. Another innovation reported in this paper is the co-encapsulation of a brominated carbocyanine dye (BrCy112) with an inorganic magnetic resonance (MR) contrast agent to potentially enable dual optical and MR imaging (MRI) by these RBC nano-ghosts.

Gadolinium (a rare earth metal element) as an MR contrast agent and ICG have been assembled into hybrid nanoparticles^62^ or encapsulated into liposomes or silicon dioxide matrices.^63^^–^^65^ Iron oxide nanoparticles have been encapsulated into RBCs and nano-sized RBC-derived vesicles.^66^^,^^67^ However, co-encapsulation of a brominated NIR fluorophore with an MR contrast agent into RBC nano-ghosts, presented in this study, is new. Specifically, we have co-encapsulated gadobenate dimeglumine (Gd-BOPTA, MW = 1058.17) (MultiHance™), which consists of the paramagnetic gadolinium ion chelated with benzyloxypropionictetra-acetate (BOPTA).^68^ Gd-BOPTA is an FDA-approved agent for use in MRI of the central nervous system in adults and pediatric patients and MR angiography of renal and aorto-iliofemoral occlusive vascular disease.^69^^–^^73^ The RBC nano-ghosts with dual MR and NIR fluorescence imaging capabilities can potentially be useful in surgical applications where the MRI component can provide deep tissue imaging on the order of >10  cm, and the NIR fluorescence component would allow imaging of the superficial structures at cellular and sub-cellular resolutions. As an example, for image-guided resection of epithelial ovarian tumors, which account for more than 85% of ovarian tumors,^74^ MRI can be used for pre-surgical planning to stage and map the tumor’s distribution, and fluorescence imaging would enable intraoperative imaging to visualize small tumor nodules (e.g., <1  mm) that may not otherwise be detectable for resection.^65^

## 2 Methods

### 2.1 Fabrication of RBC Nano-Ghosts Containing ICG or Various Brominated Cyanine Dyes

Erythrocytes were isolated from whole human blood (BioIVT, Westbury, New York, United States) and washed three times in isotonic (∼320  mOsm) PBS (Fisher Scientific, Hampton, New Hampshire, United States), referred to as 1X PBS, at 1000g for 10 min at 4°C. Isolated erythrocytes were then incubated with hypotonic PBS (∼80  mOsm; 0.25X PBS) at 4°C for 1 h, followed by centrifugation at 20,000g for 20 min at 4°C. The resulting hemoglobin-depleted micro-sized erythrocyte ghosts (μEGs) were resuspended in 1X PBS. To obtain nano-sized EGs (nEGs), μEGs were diluted 1:10 in 1X PBS prior to the extrusion process. The diluted μEGs were extruded three times sequentially through 800-, 400-, and 200-nm polycarbonate porous filters (Sterlitech Corp., Kent, Washington, United States) using a 10-mL automatic LIPEX^®^ extruder (Transferra Nanosciences Inc., Burnaby, British Columbia, Canada). The resulting nEGs were then centrifuged at 100,000g for 1 h at 4°C, resuspended in 1X PBS, and concentrated back by 10 times.

The brominated carbocyanine dyes, BrCy106, BrCy111, and BrCy112 (NanoQuantum Sciences, Bellevue, Washington, United States) and ICG (MP Biochemicals, Santa Ana, California, United States) were used to form variants of RBC nano-ghosts. We incubated the nEG solution with Sørensen’s phosphate buffer (${\mathrm{Na}}_{2}{\mathrm{HPO}}_{4}/{\mathrm{NaH}}_{2}{\mathrm{PO}}_{4}$, ∼140  mOsm, pH∼8) and solutions of each of the dyes, previously dissolved in water, in a 1:1:1 volume ratio for 30 min at 4°C to form the RBC nano-ghost variants BrCy106-nEGs, BrCy111-nEGs, BrCy112-nEG, and ICG-nEGs. The resulting pellets were then washed twice in 1X PBS and centrifuged at 100,000g for 1 h at 4°C to remove excess unencapsulated dyes. We experimented with dye concentrations of 100, 750, and 1000  μM in the loading solution. Triplicate RBC nano-ghost samples loaded with each of the dyes were prepared.

### 2.2 Fabrication of RBC Nano-Ghosts Containing Gd-BOPTA and BrCy112 (Gd-BOPTA-BrCy112-nEGs)

To load the nEGs with Gd-BOPTA (Bracco Diagnostics Inc., Princeton, New Jersey, United States) and BrCy112, we incubated the nEG solution with Sørensen’s phosphate buffer (${\mathrm{Na}}_{2}{\mathrm{HPO}}_{4}/{\mathrm{NaH}}_{2}{\mathrm{PO}}_{4}$, ∼140  mOsm, pH∼8) containing both agents [Gd-BOPTA (final concentration of 125 mM) and BrCy112 (final concentration of 100  μM) in a 1:1:1:1 volume ratio for 30 min at 4°C], followed by washing in 1X PBS twice. The resulting pellets were resuspended in 1X PBS. We refer to these RBC nano-ghosts as Gd-BOPTA-BrCy112-nEGs.

### 2.3 Characterizations of Non-encapsulated Dyes and RBC Nano-Ghosts

The absorption spectra of non-encapsulated (free) BrCy106, BrCy111, BrCy112, ICG, and the various RBC nano-ghosts in 1X PBS were obtained using a spectrophotometer (Jasco V-670 UV-vis spectrophotometer, Jasco Inc., Easton, Maryland, United States) with an optical path length of 1 cm. The slit size for the spectrofluorometric measurements was 5 nm. Free dyes were initially dissolved in 1X PBS at concentrations of 100, 750, and 1000  μM because these were the concentrations used in fabricating the optical RBC nano-ghosts. For spectrometric recordings, the free dye solutions were diluted 20 times to avoid detector saturation, hence resulting in concentrations of 5, 37.5, and 50  μM in PBS during the measurements. The suspensions of RBC nano-ghosts in 1X PBS were diluted by 10 times prior to spectral recordings.

Fluorescence emission spectra of 10X-diluted RBC nano-ghosts and 20X-diluted free dyes in response to photoexcitation at 750±2.5  nm (for brominated dyes and BrCy-loaded nEGs) and 780±2.5  nm (for free ICG and ICG-loaded nEGs) filtered from a 450-W xenon lamp were acquired using a fluorometer (Fluorolog-3 spectrofluorometer, Horiba Jobin Yvon Inc., Edison, New Jersey, United States). We obtained the normalized spectrally integrated fluorescence emission σ as

$$\sigma =\frac{\int F(\lambda )d\lambda_{\mathrm{em}}}{1-10^{-A(\lambda_{\mathrm{ex}})}},$$ (1)

where F is the wavelength-dependent fluorescence emission intensity in response to excitation wavelength ($\lambda_{\mathrm{ex}}$), and A is the absorbance of the sample at the excitation wavelength. Fluorescence emission was integrated over the range of 765 to 900 nm for BrCy-nEGs and 795 to 900 nm for ICG-nEGs.

We determined the relative fluorescence quantum yield ϕ for each of the free BrCy dyes and RBC nano-ghost variants in response to 750-nm (for BrCy-encapsulating NPs) or 780-nm (for ICG-encapsulating NPs) excitation wavelength as

$$\phi_{\text{sample}}=\phi_{\mathrm{ICG}}\times \sigma_{\text{sample}}\times [\frac{1-10^{-A_{\mathrm{ICG}}(\lambda_{\mathrm{ex}}=780\;\mathrm{nm})}}{\int F_{\mathrm{ICG}}(\lambda_{\mathrm{ex}}=780\;\mathrm{nm})d\lambda_{em}}],$$ (2)

where $\phi_{\mathrm{ICG}}$ is the fluorescence quantum yield of free ICG (∼1.7%) in PBS at 779-nm excitation wavelength,^75^^,^^76^ $\sigma_{\text{sample}}$ is the normalized spectrally integrated fluorescence emission of free BrCy dyes or RBC nano-ghosts in response to a given excitation wavelength, $A_{\mathrm{ICG}}$ is the absorbance value of free ICG at 780 nm, and the integral represents the spectrally integrated fluorescence emission of free ICG in response to 780-nm excitation wavelength.

We define the loading efficiency of each dye into the RBC nano-ghosts as

$$\text{Loading Efficiency}=1-\frac{m_{\text{super}}}{m_{\text{initial}}},$$ (3)

where $m_{\text{super}}$ is the amount of the dye remaining in the supernatant upon completing the fabrication of the RBC nano-ghosts, and $m_{\text{initial}}$ is the amount of the dye introduced into the loading buffer. To determine the amount of dye in the supernatant, we measured the absorbance value of the supernatant solution and then compared the peak absorbance value of the supernatant to a calibration curve that related the peak absorbance value at the same wavelength to various known concentrations of ICG or the BrCy dye in the same supernatant buffer.

The hydrodynamic diameter distributions for all RBC nano-ghosts suspended in 1X PBS were measured by dynamic light scattering (DLS) (Zetasizer Nanoseries, NanoZS90, Malvern Panalytical Ltd., Malvern, United Kingdom). Three individual measurements were collected for each sample, averaged, and standard deviation (SD) determined for each measured diameter. The zeta potentials (Zetasizer Nanoseries, NanoZS90, Malvern Panalytical Ltd.) of the RBC nano-ghosts suspended in folded capillary cells were measured in 1X PBS. Five measurements were collected for each sample and averaged to determine the mean ± SD values of the zeta potential for each sample type.

### 2.4 Magnetic Resonance Imaging

MRI was performed using a 3T Siemens PRISMA scanner (Siemens Medical Solutions, Inc., Forchheim, Germany). The T1 values of the contrast agent (Gd-BOPTA) were measured with a spin echo–based inversion recovery experiment (repetition time = 5000 ms; echo time = 15 ms, field of view=210  mm×72  mm, matrix size=256×88). Inversion times (TI) were 30, 100, 400, 1000, 2000, 3000, 4000, and 4900 ms. T1 values were calculated voxelwise by fitting the resulting signal intensity (S) curve with an exponential function

S(t)=A−B exp(−TI/T1). (4)

### 2.5 Quantification of Gd-BOPTA and Fluorophores Loading Efficiency into nEGs

To quantify the loading efficiency of Gd-BOPTA, we used a similar methodology as described above [Eq. (3)]. We obtained the MR images of the particles and the supernatants collected upon completing the fabrication of Gd-BOPTA-BrCy112-nEGs. To quantify the amount of Gd-BOPTA in the supernatant, we prepared various concentrations of the Gd-BOPTA (10, 30, 60, 125, 180, and 250 mM) in water and measured the T1 relaxation rate (1/T1 = R1) of these suspensions diluted 100 times to generate a standard curve of T1 value versus concentration. By comparing to the standard curve, we obtained the concentrations of non-encapsulated Gd-BOPTA in the supernatant after fabrication of Gd-BOPTA-BrCy112-nEGs.

### 2.6 Assessment of BrCy112 and Gd-BOPTA Leakage from Gd-BOPTA-BrCy112-nEGs

Upon fabricating the Gd-BOPTA-BrCy112-nEGs, the suspension containing the particles was stored at 4°C. At specific times (0, 2, and 7 days), ∼1.3  mL of particle suspension was centrifuged, and the pellet was resuspended in the original volume of 1X PBS. We recorded the absorption and the fluorescence emission spectra of the resuspended pellet and supernatant. To determine BrCy112 leakage, we compared the absorption and fluorescence emission spectra and their spectrally integrated emission from days 2 and 7 to day 0. We also measured the T1 values of non-diluted and 100 time-diluted Gd-BOPTA-BrCy112-nEGs at these time points to determine the Gd-BOPTA leakage by comparing their corresponding R1 values at days 0, 2, and 7. Three measurements were obtained for each time point and averaged to determine the mean R1 value. All characterization results reported in this study were obtained at room temperature.

### 2.7 Cytotoxicity Studies

We cultured SKOV3 ovarian cancer cells in 96-well plates containing McCoy’s 5A medium supplemented with 10% fetal bovine serum and 1% antibiotics for 24 h in advance of cytotoxicity assessment to ensure adhesion and cell density growth to $∼10^{6}\text{ cells}/\mathrm{mL}$. On the following day, cells were washed with sterile PBS and then incubated with free BrCy112 (50  μM) or BrCy112-nEGs (fabricated using 100  μM of BrCy112 in the loading buffer) in the culture medium for 3 and 24 h. We chose BrCy112 as the test dye for the cytotoxicity studies because it was the same dye used in fabricating the nEGs with dual optical and MR characteristics. Cells incubated with the complete culture medium for 24 h without any additional reagents were used as the positive control population. Cells incubated with 100  μL methanol for 24 h were used as the negative control population.

Cells were washed twice with sterile PBS post-incubation with free BrCy112 and BrCy112-nEGs and stained using the live/dead cytotoxicity kit (L3224, Invitrogen, Waltham, Massachusetts, United States). This kit discriminates the live cells from dead cells by simultaneous staining with green-fluorescent Calcein AM to assay intracellular esterase activity and red-fluorescence ethidium homodimer-1 to indicate loss of plasma membrane integrity. We used a filter passing green fluorescence from the live cells over the spectral band of 514 to 560 nm in response to the excitation light in the range of 463 to 600 nm filtered from a xenon lamp, and a filter passing the red fluorescence from the dead cells in the range of 590 to 650 nm when photoexcited in the range of 528–555 nm. An EM-CCD camera (C9100-14, Hamamatsu, Japan) with integration times in the range of 30 to 50 ms at unity gain was used to capture the fluorescence emission. To quantify the percentage of live cells, green and red stained cells were counted separately by the automated cell counter in ImageJ. After threshold adjustment, the ImageJ built-in watershed feature was applied on each image to separate any overlapping cells. Afterward, ImageJ analyzed the images and generated auto-counted cell numbers.

## 3 Results and Discussion

### 3.1 Absorption and Fluorescence Characteristics of Free Dyes

Absorption spectra of free BrCy and ICG fluorophores dissolved in 1X PBS at three different concentrations (5, 37.5, and 50  μM) are shown in Fig. 2. The spectra of free BrCy106 showed a narrow distinct spectral peak at ∼750  nm [Fig. 2(a)], which we attribute to the monomeric form of the dye.^33^ As the concentration of BrCy106 increased from 5 to 37.5 and 50  μM, a more visible shoulder in the range of 671 to 700 nm emerged, which can be associated with an H-like aggregate form of the dye.^77^^,^^78^ In such aggregates, fluorophores are stacked in a sandwich-like structure.^79^ In accordance with the exciton theory,^80^ aggregates split the energy level of the excited state into two new nondegenerative states that have two different energy levels. In H-like aggregates, transition only to the upper excited states is allowed; hence, the absorption spectrum includes a band (671 to 700 nm in the case of BrCy106), which is blue-shifted (i.e., a hypsochromic shift) with respect to the monomer peak at 750 nm.

As the concentration of free BrCy111 in 1X PBS was increased to 37.5 and 50  μM, bimodal spectra with respective primary and secondary peaks at 850 and 643 nm, respectively, and a notably broadened spectrum emerged [Fig. 1(b)]. The bathochromic (red) shift to 850 nm is consistent with the presence of J-like aggregates of the dye. In such aggregates, the dye molecules are arranged in a head-to-tail manner, and transition only to the lower level of the split excited state is allowed.^81^ Therefore, the spectral peak of the absorption of the aggregate is red-shifted relative to the absorption of the monomer. We also point out that in addition to the type of packing (sandwich-like or head-to-tail), the difference in the structural packing of the chromophores in H- and J-like aggregates is also a result of the different slip angles of the molecules where the slip angle is defined as the angle between the transition dipoles and the molecular axis of the aggregate.^79^

In comparison with BrCy106 and BrCy112, BrCy111 has two dibromobenzene rings (Fig. 1). The fact that BrCy111 is more extensively brominated combined with the chemical composition of PBS can make BrCy111 more prone to aggregation. Specifically, with increased bromination, the role of hydrophobic driving forces, including London dispersion forces (induced dipole–induced dipole interactions) for aggregation, increases.^77^ The increased hydrophobicity can be the result of enhanced electron-withdrawing effects by the additional bromine atoms, which due to their electronegativity can draw electrons from the carbon atoms to which they are bonded and create partially positive charges around them.^82^ Such partially positive-charged portions increase the affinity of the dye for less polar and non-polar environments, leading to increased hydrophobicity.^83^ Furthermore, electron-withdrawing effects allow the energy levels of the dye to be adjusted over a broad range.^84^^,^^85^

The symmetry of BrCy111 can also make it more susceptible to aggregation. Since fundamentally the formation of aggregates is driven by intermolecular electrostatic interactions, the symmetry can promote stronger intermolecular interactions, leading to stackings (e.g., π–π stacking),^86^^–^^88^ particularly resulting from the additional symmetric bromination in the case of BrCy111, dipole–dipole interactions, and London forces that ultimately alter the electronic energy levels of the dye.

The ionic strength of the solvent is also a contributing factor to the propensity of the dye molecules to aggregate.^77^ For example, the presence of salts (NaCl, KCl, ${\mathrm{Na}}_{2}{\mathrm{HPO}}_{4}$, and ${\mathrm{KH}}_{2}{\mathrm{PO}}_{4}$) in PBS contributes to the formation of J-aggregates.^89^ The salt ions reduce the electrostatic repulsions among the dye molecules, thereby accelerating the formation of aggregates.^90^^,^^91^ In summary, we attribute the observed absorption spectra of free BrCy111 to the formation of dye aggregates, resulting from the additional bromination, which increases the hydrophobic forces among the dye molecules, the symmetry of the dye, and the effects of the salt ions in PBS, which further enhance the strength of aggregation among the dye molecules.

As the concentration of free BrCy112 was increased to 37.5 and 50  μM in 1X PBS [Fig. 2(c)], the absorption spectra showed a distinct spectral peak at 744 nm, corresponding to the monomer form of the dye, and shoulders in the range of 668 to 697 nm and 790 to 825 nm, suggesting the simultaneous presence of both the H-like and J-like aggregates, respectively. For free ICG dissolved in 1X PBS [Fig. 2(d)], the peak at 780 nm corresponds to its monomeric form. Increasing the ICG concentrations to 37.5 and 50  μM was associated with the emergence of another peak at 706 nm, attributed to the H-like aggregate form of the dye.^92^ For all four dyes, the increase in absorbance values did not scale linearly with the change in the dye concentration from 37.5 to 50  μM. These results indicate that the range of the linear relationship between absorbance and dye concentration is below 37.5  μM for these dyes when dissolved in isotonic PBS.

**Fig. 3:**
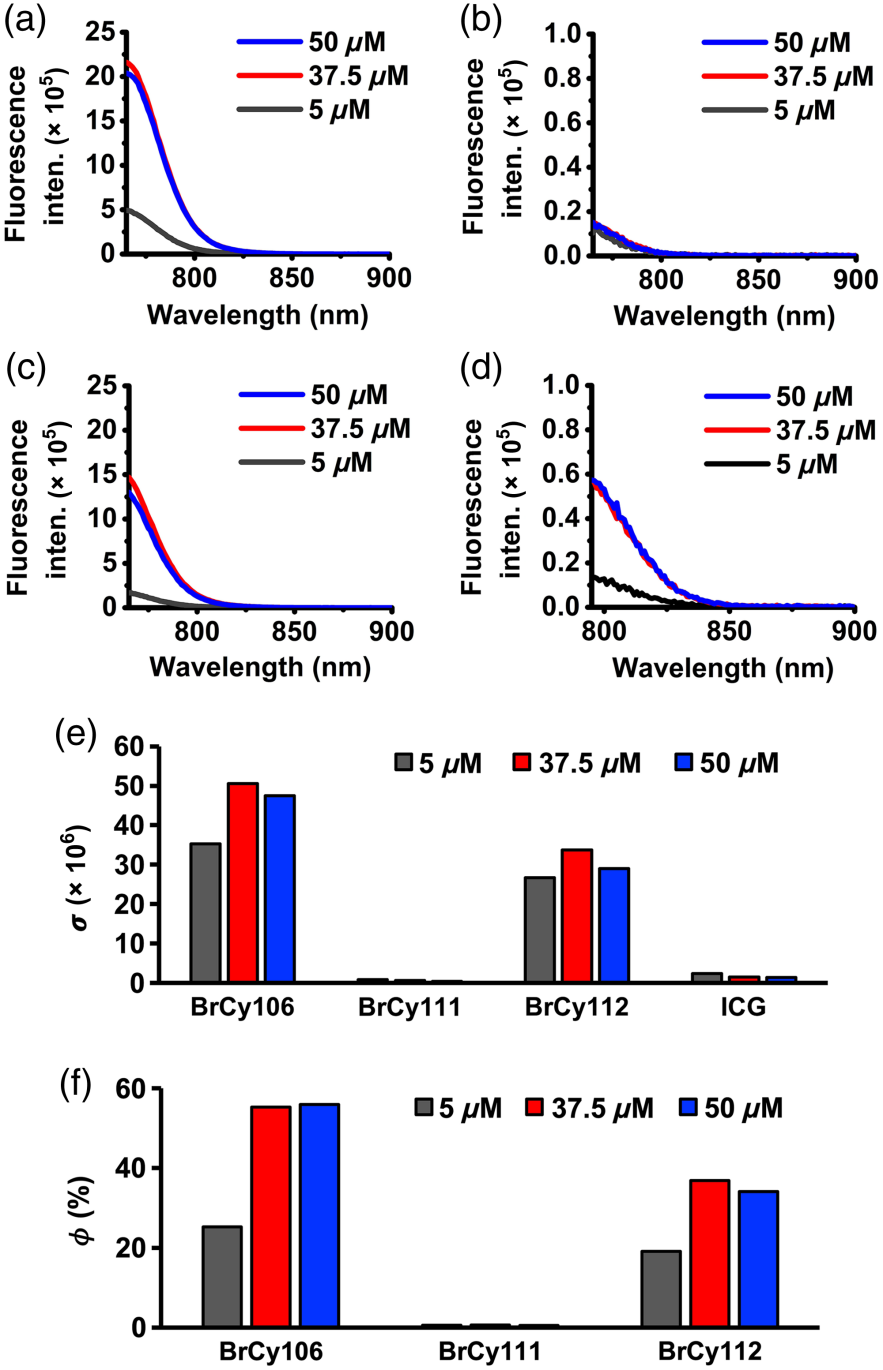
(a)–(d) Fluorescence emission spectra of free BrCy106 (a), BrCy111 (b), BrCy112 (c), and ICG (d) in 1X PBS in response to photoexcitation wavelengths of 750±2.5  nm (for BrCy dyes) and 780±2.5  nm (for ICG). (e) Normalized spectrally integrated fluorescence (σ) over the 765 to 900 nm spectral band for free BrCy dyes (excited at 750 nm) and 700 to 900 nm spectral band for ICG (excited at 780 nm). (f) Relative fluorescence quantum yield (ϕ) for the three free BrCy dyes as compared with free ICG in 1X PBS.

In response to photoexcitation of free BrCy dyes at 750±2.5  nm and ICG at 780±2.5  nm, the peak values of the fluorescence emission intensities for free BrCy106 and BrCy112 [Figs. 3(a) and 3(c)] were considerably greater than those for free BrCy111 and ICG [Figs. 3(b) and 3(d)]. For example, for BrCy106 and BrCy112 at 37.5  μM, the peak emission intensities were respectively 38 and 26 times higher as compared with free ICG. Similarly, the values of the normalized spectrally integrated fluorescence (σ) were considerably greater for free BrCy106 and BrCy112 [Fig. 3(e)]. Both free BrCy106 and BrCy112 had the highest values of σ at 37.5  μM. At this concentration, the value of σ associated with free BrCy106 was ∼33 times higher as compared with the value for free ICG prepared at the same concentration [Fig. 3(e)]. Increasing the concentrations of free BrCy111 and ICG from 5 to 37.5 and 50  μM was associated with progressively lowered values of σ, suggestive of dye aggregation-induced weakening of fluorescence.^16^

At each of the experimented concentrations, free BrCy106 produced the highest value of the relative fluorescence quantum yield (ϕ) [Fig. 3(f)]. For example, although ϕ for free BrCy106 nearly doubled when increasing the concentration from 5 to 37.5  μM, there was not much further increase in its ϕ with increased concentration to 50  μM. These results are in line with the results shown in Figs. 3(a)–3(e) where there are minimal changes in the emission intensities and σ when concentrations of the dyes in PBS are greater than 37.5  μM. The lower values of ϕ for free BrCy112 as compared with BrCy106 are suggestive of the greater aggregated forms of free BrCy112, evidenced by the presence of hypsochromic and bathochromic shifts in its absorption spectrum [Fig. 2(c)]. The minimal values of ϕ for free BrCy111 (in the range of 0.5% to 0.7% depending on the concentration) can be attributed to aggregation-induced fluorescence quenching, indicating that the excited state energy is mostly dissipated through non-radiative relaxation pathways.^93^

### 3.2 Characterizations of BrCy- and ICG-Encapsulating Nano-Sized Erythrocyte Ghosts (EGs)

**Fig. 4:**
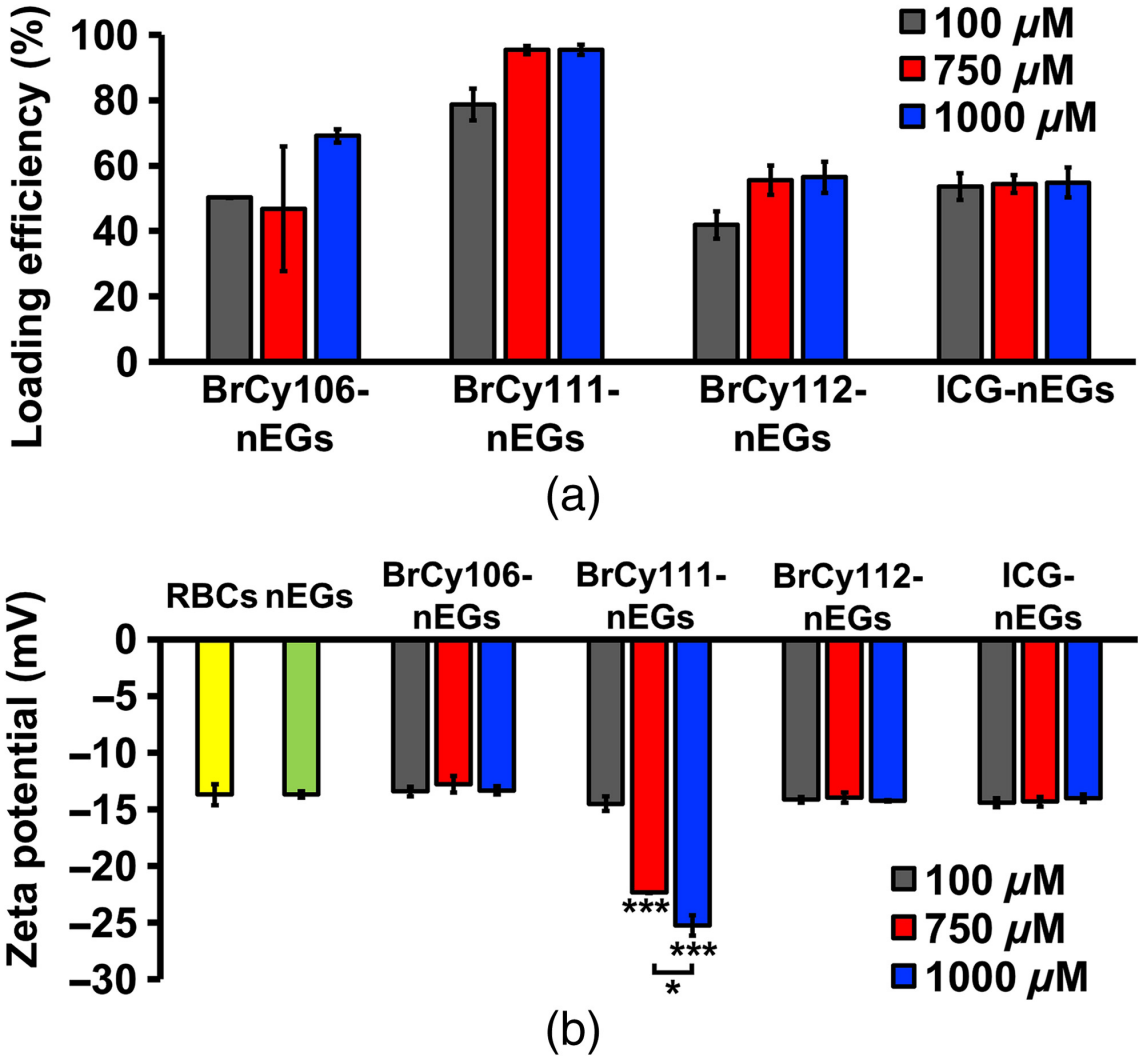
(a) Mean loading efficiency of various BrCy dyes and ICG into nano-sized EGs. (b) Mean zeta potentials for RBCs, nEGs, and BrCy- and ICG-encapsulating nano-sized EGs suspended in 1X PBS. Single asterisk (*) denotes a statistically significant differences (p<0.05) between the indicated pair. Triple asterisks (***) for BrCy111-nEGs fabricated at 750 and 1000  μM indicate that those mean zeta-potential values were significantly different (p<0.001) as compared to the values for other nanoparticles.

At a given concentration used to fabricate the particles, BrCy111 had the highest loading efficiency, in the range of ∼79% to 95% [Fig. 4(a)]. The high loading efficiency of BrCy111 is suggestive of its favorable interactions (e.g., stronger hydrophobic interactions) with the membrane of the EGs. However, despite its relatively high loading efficiency, BrCy111-nEGs exhibited inferior fluorescence characteristics as compared with the nEGs loaded with BrCy106 or BrCy112 (Fig. 7). For BrCy106, its mean loading efficiency was increased by ∼48% when its concentration in the loading buffer was increased from 750 to 1000  μM, whereas for BrCy111 and BrCy112, this change in concentration was not accompanied by an increase in loading efficiency. In the case of ICG, its loading efficiency remained at ∼54% independent of its concentration in the loading buffer.

**Fig. 5:**
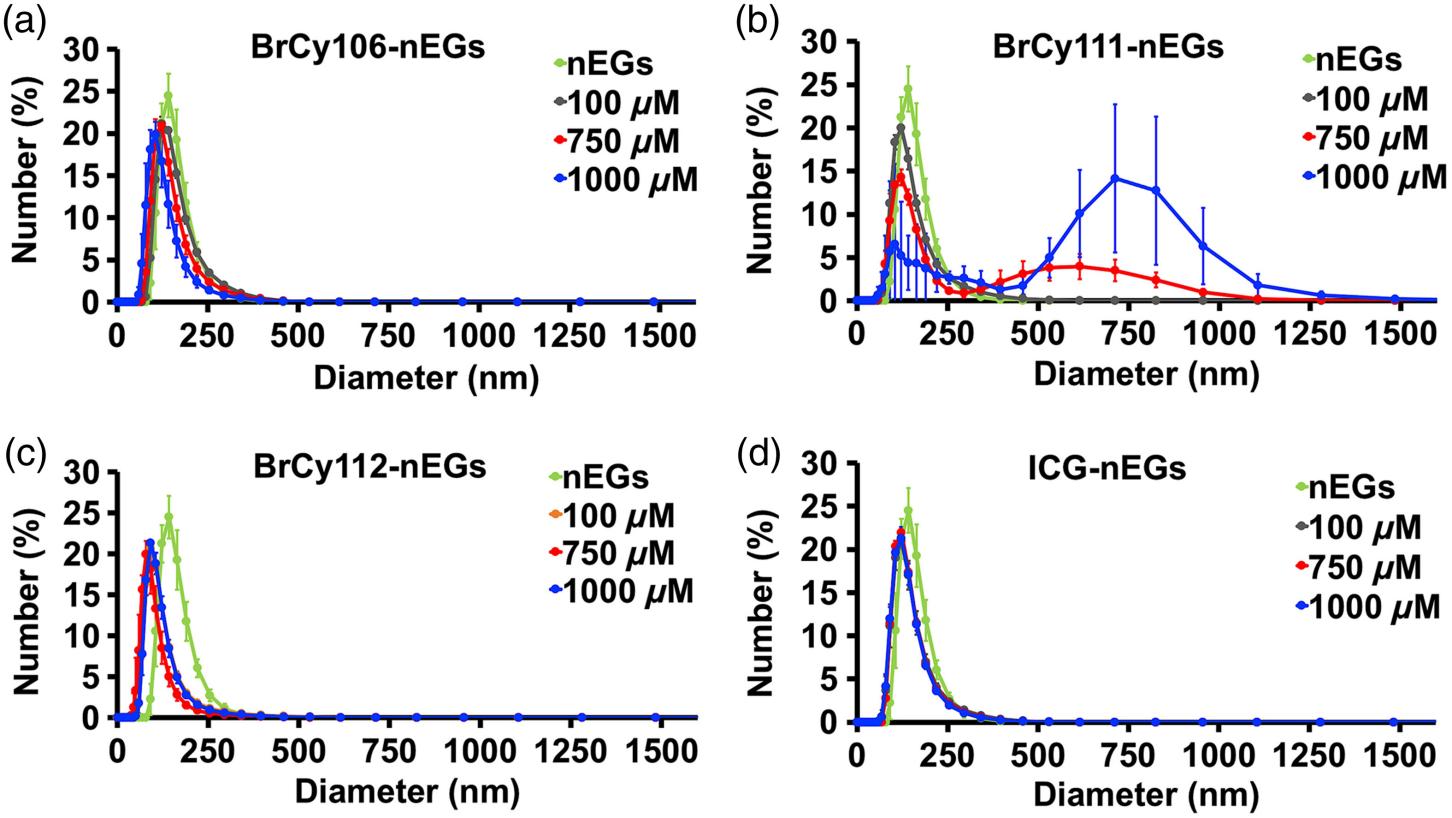
Hydrodynamic diameter distributions for nEGs loaded with various BrCy dyes or ICG measured in 1X PBS by DLS. (a) BrCy106-nEGs. (b) BrCy111-nEGs. (c) BrCy112-nEGs. (d) ICG-nEGs.

Except for BrCy111-nEGs, which had the respective mean zeta potential values of −22.34 and −25.25  mV when fabricated using 750 and 1000  μM of the dye, the remaining RBC nano-ghosts had mean zeta potentials in the range of −12.79 to −14.43  mV [Fig. 4(b)]. This range was similar to the mean values for nEGs (−13.68  mV) and untreated RBCs (−13.7  mV). The more negative zeta potential for BrCy111-nEGs may be a result of the presence of BrCy111-nEG aggregates when using the higher concentrations of 750 and 1000  μM in fabricating the particles. Given the larger surface area of the aggregates, there could be an increased number of exposed negatively charged species (e.g., sulfonates) to cause a more negative value of the zeta potential. Further evidence in support of the formation of BrCy111-nEG aggregates is shown in Fig. 5(b).

Illustrative diameter distributions for RBC nano-ghosts loaded with various dyes, acquired by DLS, are shown in Fig. 5. For a given RBC nano-ghost type, there were minimal variations in the diameter distribution and the mean peak diameter as a function of the dye concentration used in fabricating that RBC nano-ghost [Figs. 5(a), 5(c), and 5(d)], with the exception of BrCy111-nEGs fabricated at 750 and 1000  μM, which exhibited bimodal distributions [Fig. 5(b)]. We attribute the right-shifted distributions (population distributions with larger diameters) for the BrCy111-nEGs to the aggregates of these particles. Such aggregates can be formed as a result of attractive forces (e.g., van der Waals forces) among the BrCy111-nEGs if the BrCy111 molecules, which themselves are aggregated [as supported by the absorption spectra shown in Fig. 2(b)] and are not fully encapsulated. For example, the exposed portions of the non-encapsulated BrCy111 molecules may consist of the negatively charged sulfonates that would have electrostatic interactions with the positively charged hydrogens of the sulfonic acid groups protruding out from BrCy111 molecules on other BrCy111-nEGs. The remaining RBC nano-ghosts had mean peak diameters in the range of 84 to 137 nm.

**Fig. 6:**
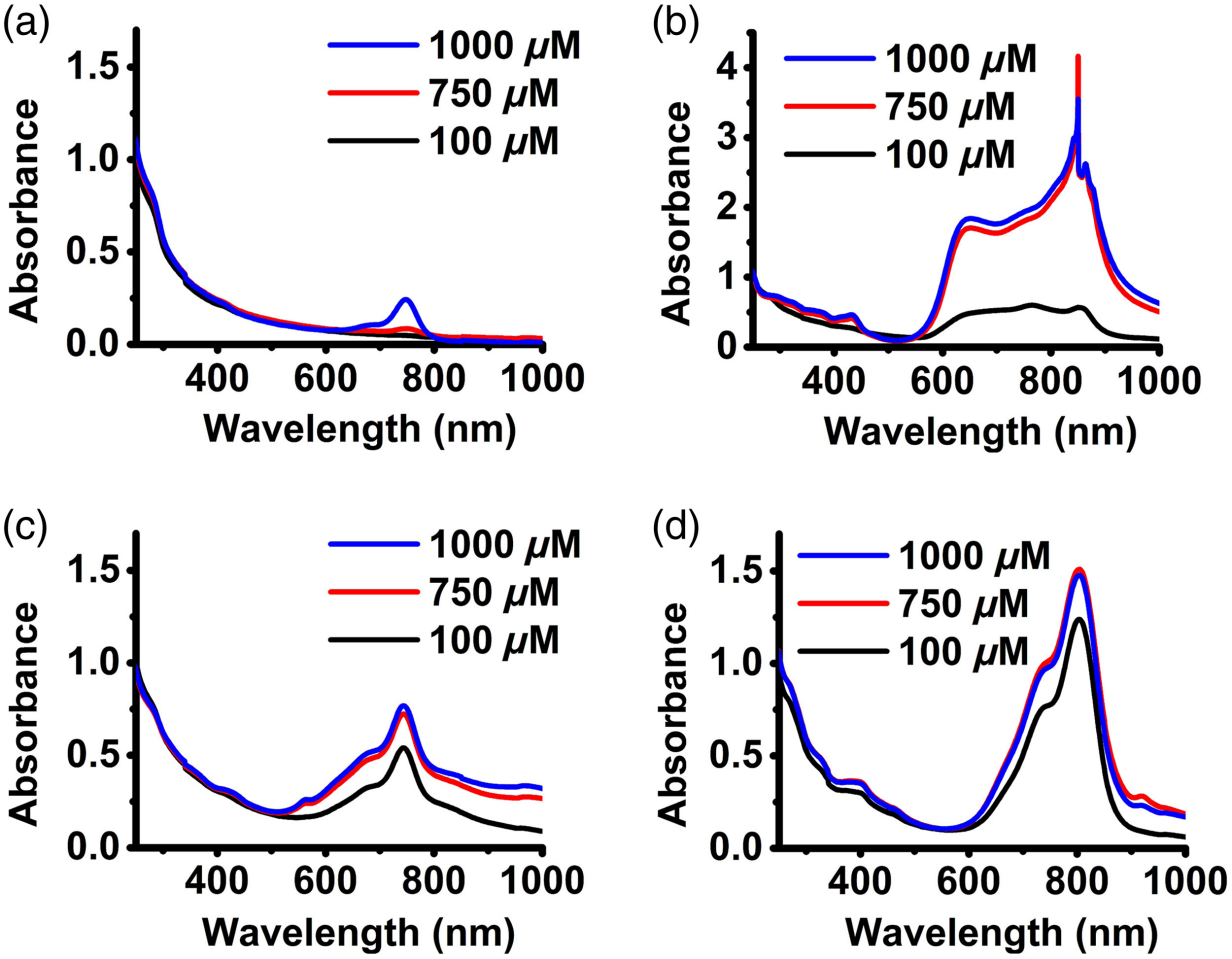
Absorption spectra of various optical RBC nano-ghosts suspensions in 1X PBS. (a) BrCy106-nEGs. (b) BrCy111-nEGs. (c) BrCy112-nEGs. (d) ICG-nEGs.

Absorption spectra of the RBC nano-ghosts doped with the various dyes are shown in Fig. 6. Absorbance at 280 nm originates from the aromatic amino acids of the RBC proteins such as tyrosine and tryptophan.^94^^–^^96^ The absorbance values at 280 nm for all RBC nano-ghosts was ∼1, indicating that the constructs had approximately the same amount of membrane materials. For BrCy106-nEGs, as the dye concentration used in fabricating the particles was increased to 1000  μM, the spectral peak at 750 nm, associated with the monomer form of BrCy106, emerged [Fig. 6(a)]. As the BrCy111 concentration used in fabricating the BrCy111-nEGs was increased from 100 to 750 and 1000  μM, the monomer peak at 854 nm was no longer distinct [Fig. 6(b)]. Hypsochromic and bathochromic shifts with peaks at 652 and 854 nm, respectively, and a broadened spectrum, similar to the features for free BrCy111 [Fig. 2(b)], were observed, suggesting that the dye was mostly in the forms of H-like and J-like aggregates when encapsulated. The spikes associated with the spectra of BrCy111-nEGs fabricated using the higher concentrations of the dye (750 and 1000  μM) are possibly due to instrumentation artifacts or the sample impurities.

The absorption spectra of BrCy112-nEGs [Fig. 6(c)] were also similar to those for free BrCy112 [Fig. 2(c)] with a distinct peak at 744 nm and shoulders in the range of 661 to 697 nm and 797 to 850 nm, which can be attributed to the monomer form, H-like, and the J-like aggregates of the dye, respectively. The ICG-nEGs exhibited a primary peak at 804 nm and a shoulder in the range of 727 to 758 nm, associated with the monomer and the H-like aggregate forms of the dye, respectively. Consistent with our previous results,^46^ the monomeric peak had a bathochromic shift from 780 nm as compared with free ICG, indicative of altered electronic states of ICG when nano-encapsulated. As the ICG concentration used in fabricating the ICG-nEGs was increased to 750 and 1000  μM, another peak at 919 nm emerged, suggestive of the formation of J-like aggregates.

**Fig. 7:**
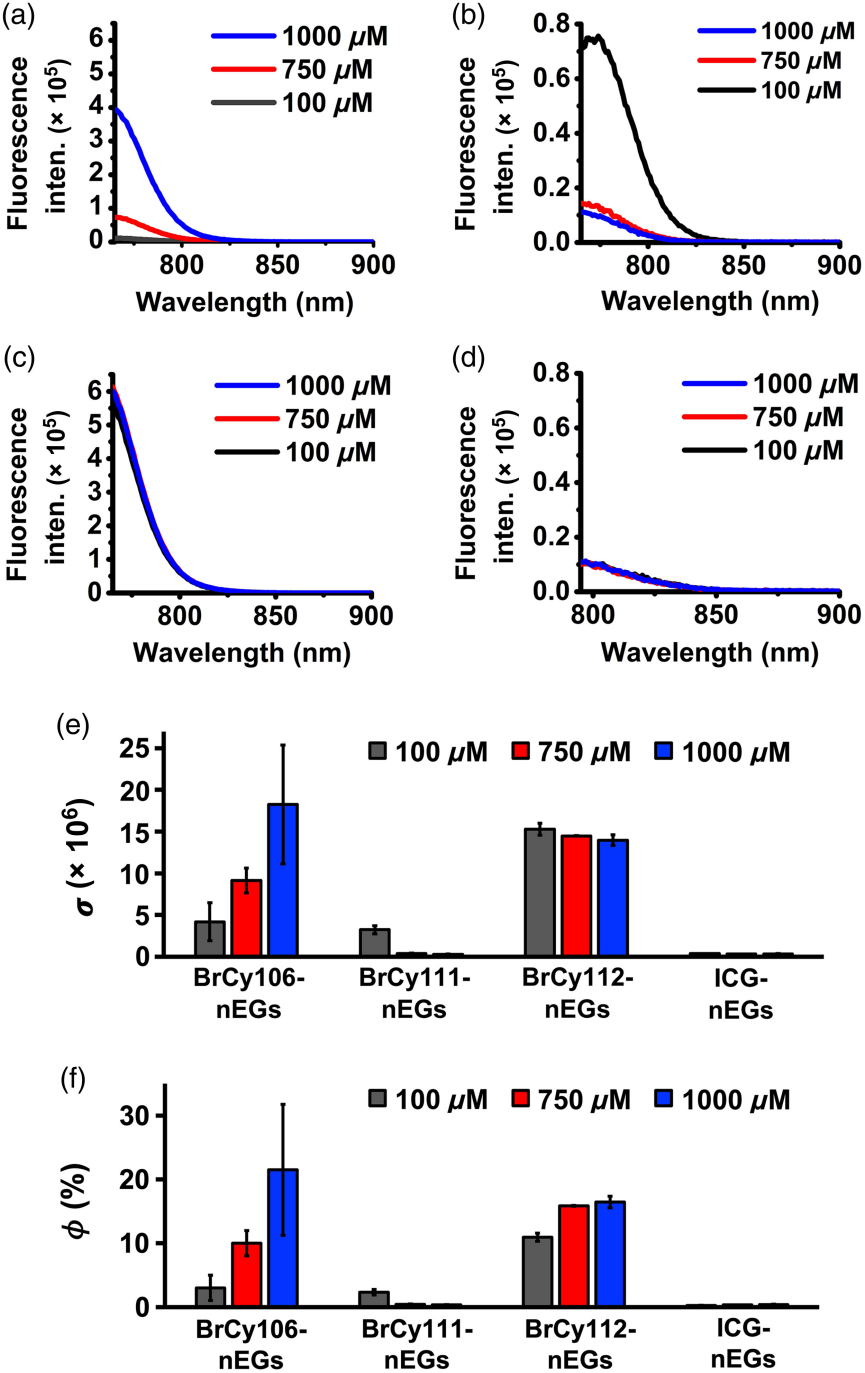
Fluorescence emission spectra of (a)–(c) BrCy106-nEGs, BrCy111-nEGs, and BrCy112-nEGs in response to photoexcitation at 750±2.5  nm and (d) ICG-nEGs in response of photoexcitation at 780±2.5  nm. (e) Normalized spectrally integrated fluorescence values (σ) for various BrCy- and ICG-nEGs. (f) Fluorescence quantum yield (ϕ) for various RBC nano-ghosts fabricated at different concentrations of the dyes relative to that of free ICG in 1X PBS at 779 nm.

In response to photoexcitation at 750±2.5  nm for BrCy-nEGs and 780±2.5  nm for ICG-nEGs, BrCy106-nEGs and BrCy112-nEGs in general produced the highest peak emission intensities [Figs. 7(a) and 7(c)] as compared with BrCy111-nEGs and ICG-nEGs [Figs. 7(b) and 7(d)]. Increasing the concentration of BrCy106 from 750 to 1000  μM was associated with nearly a fivefold increase in the peak emission intensity of BrCy106-nEGs [Fig. 7(a)], consistent with the increased loading efficiency of the dye with this change in concentration [Fig. 4(a)]. Increasing the concentration of BrCy111 from 100  μM was associated with a decrease in the peak emission intensity of the BrCy111-nEGs [Fig. 7(b)], consistent with aggregation-induced fluorescence quenching. In agreement with the loading efficiency of BrCy112 and ICG [Fig. 4(a)], there were minimal changes in the emission spectra of BrCy112-nEGs [Fig. 7(c)] and ICG-nEGs [Fig. 7(d)] with increasing concentrations of the dyes used to fabricate these RBC nano-ghosts. However, depending on the concentration, the peak emission intensity of BrCy112-nEGs was at least 55 times higher than that of ICG-nEGs.

Consistent with the trends in the fluorescence emission spectra [Figs. 7(a)–7(d)], values of σ for BrCy106-nEGs and BrCy112-nEGs were higher than those for BrCy111-nEGs and ICG-nEGs, except when using 100  μM of BrCy111, which produced a similar value to BrCy106-nEGs fabricated at 100  μM [Fig. 7(e)]. As the value of σ for BrCy112-nEGs fabricated using 100  μm of the dye was only ∼16% lower than the highest σ, which was associated with BrCy106-nEGs fabricated at 10 times higher concentration, we chose to proceed with BrCy112-nEGs as the test construct for co-encapsulation with Gd-BOPTA.

The highest value of ϕ (∼21.5%) was associated with BrCy106-nEGs fabricated using 1000  μM of the dye whereas ICG-nEGs had the lowest values of ϕ, in the range of ∼0.27% to 0.40% [Fig. 7(f)]. For example, when fabricated using 1000  μM of the respective dye, the value of ϕ was nearly 54 times higher for BrCy106-nEGs as compared with ICG-nEGs. Although the highest value of ϕ for RBC nano-ghosts fabricated using 100  μM of a given dye was associated with BrCy112-nEGs (∼11%), the greatest rate of increase in ϕ with increased concentration beyond 100  μM was associated with BrCy106-nEGs.

### 3.3 Dual Encapsulation of NIR Fluorescent and MR Materials

**Fig. 8:**
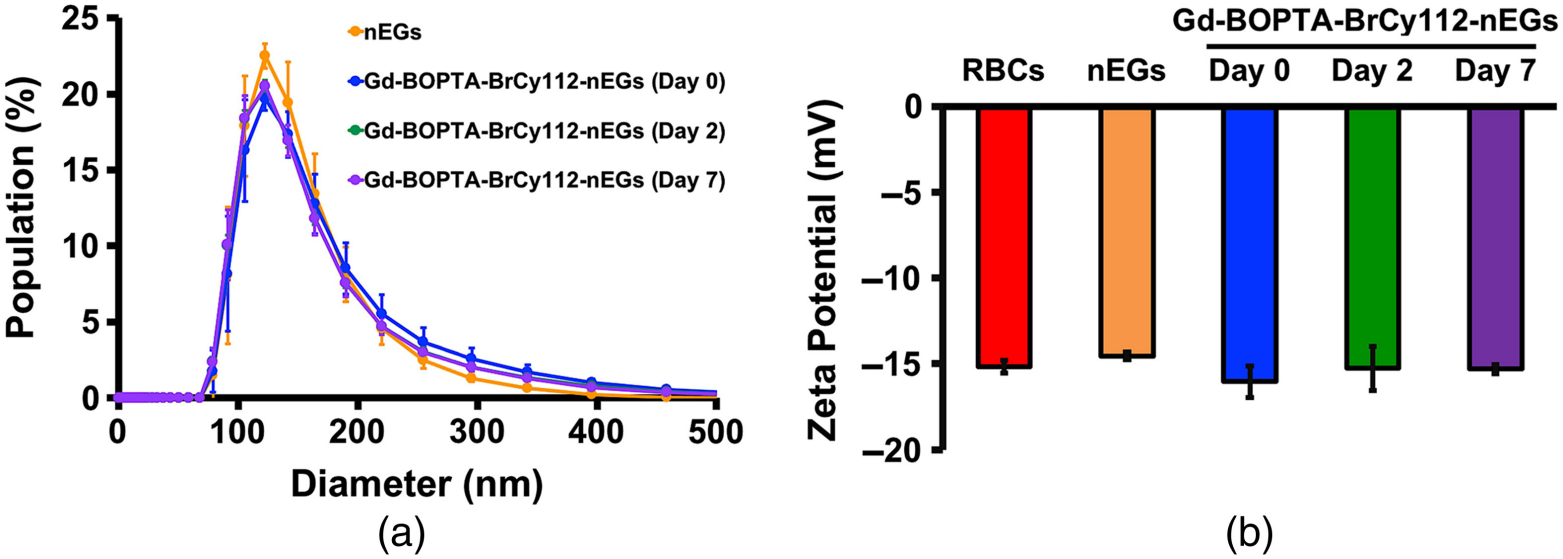
(a) DLS-based hydrodynamic diameters of nEGs and Gd-BOPTA-BrCy112-nEGs collected at day 0 (immediately after fabrication) and days 2 and 7 post-fabrication. (b) Mean zeta potentials of RBC, nEGs, and Gd-BOPTA-BrCy112-nEGs collected on days 0, 2, and 7.

To combine the strengths of NIR fluorescence imaging and MRI into a single nano-construct, we encapsulated BrCy112 and Gd-BOPTA into RBC nano-ghosts. Illustrative DLS-based estimates of nEGs and Gd-BOPTA-BrCy112-nEG diameters show a unimodal distribution, suggesting that the particles were not aggregated [Fig. 8(a)]. The mean peak hydrodynamic diameters for nEGs and Gd-BrCy112-nEGs immediately after fabrication were ∼129 and 131 nm, respectively. There were minimal changes in the diameter distributions of BOPTA-BrCy112-nEGs for up to 7 days post-fabrication, suggesting that the particles remained physically stable.

We estimated the loading efficiency of Gd-BOPTA into the particles as ∼33.6%. The mean zeta potential of Gd-BrCy112-nEGs (−16.03  mV) was similar to the values for nEGs (−14.53  mV) and RBCs (−15.17) [Fig. 8(b)], with minimal variation for up to 7 days. These results suggest that sialoglycoproteins, the primary negatively charged component of the RBC membrane^97^ and other charged structures, were retained after encapsulating BrCy112 and Gd-BOPTA into nEGs.

**Fig. 9:**
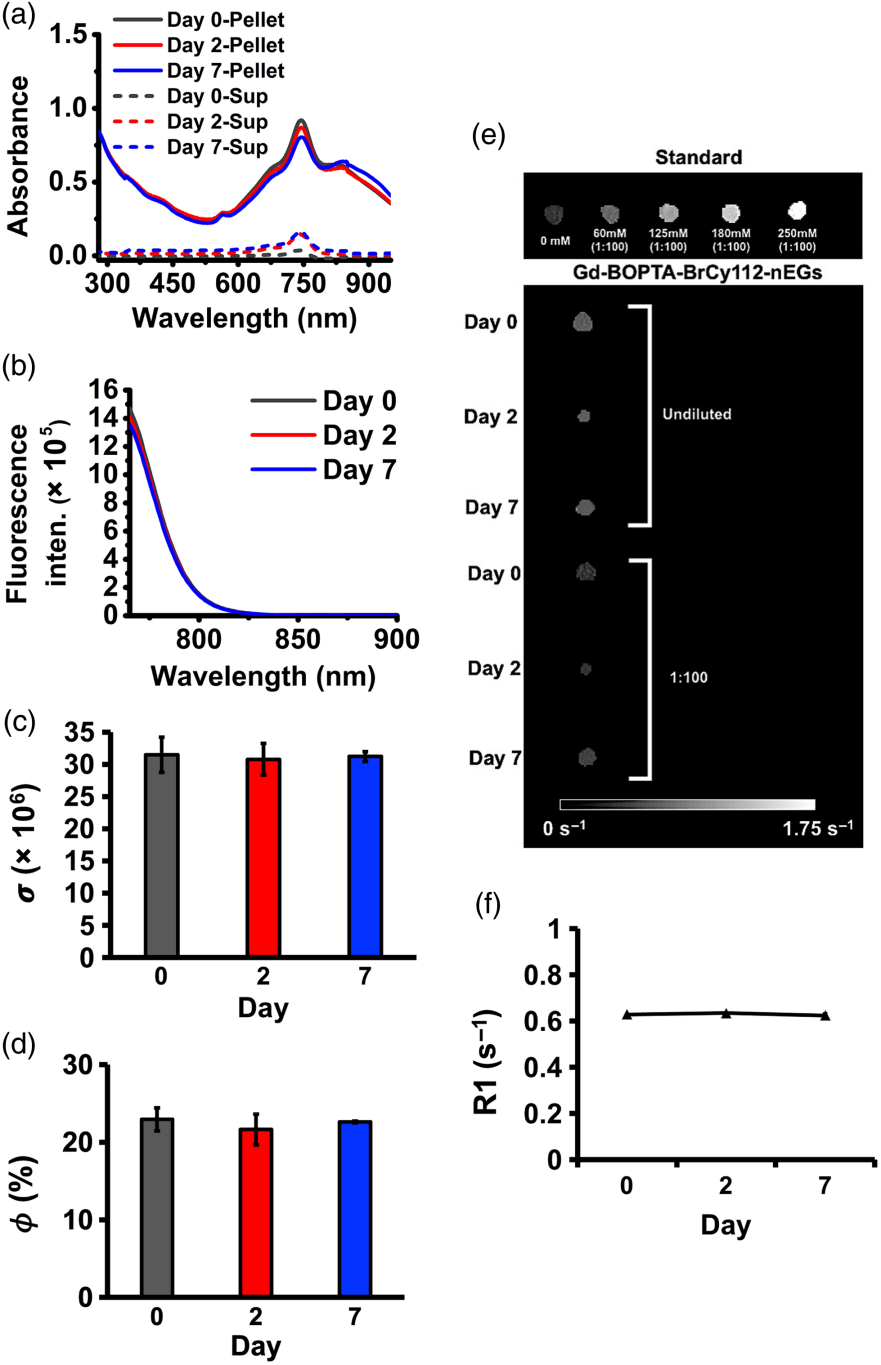
Stability results of Gd-BOPTA-BrCy112-nEGs up to 7 days post-fabrication. (a) Time-dependent absorption spectra of Gd-BOPTA-BrCy112-nEGs pellets and their corresponding supernatants (Sup) immediately after fabrication (day 0) and at days 2 and 7 of storage at 4°C. (b) Fluorescence emission spectra and (c) normalized spectrally integrated fluorescence values (σ) over the 765 to 900-nm band in response to photoexcitation at 750±2.5  nm. (d) Fluorescence quantum yield (ϕ) for Gd-BOPTA-BrCy112-nEGs at days 0, 2, and 7 relative to that of 6.45  μM free ICG in water at 780 nm. (e) MR images of Gd-BOPTA standards at different concentrations and Gd-BOPTA-BrCy112-nEGs at days 0, 2, and 7. (f) $T_{1}$ relaxation rate (R1) of Gd-BOPTA-BrCy112-nEGs. Each data point is an average of three independent measurements.

The illustrative absorption spectra of Gd-BOPTA-BrCy112-nEG pellets suspended in 1X PBS and stored for up to 7 days at 4°C, along with their corresponding supernatants are shown in Fig. 9(a). The spectra of the Gd-BOPTA-BrCy112-nEG pellets were similar to those of BrCy112-nEGs [Fig. 6(c)] featuring spectral peak at ∼745  nm with shoulders in the range of 668 to 697 and 816 to 853 nm, suggesting that in these dual-mode RBC nano-ghosts, BrCy112 molecules were still present as monomers and H-like and J-like aggregates. There was only ∼12% reduction in the monomer absorbance value at 7 days post-fabrication, concomitant by a similar increase (∼13%) in the value for the supernatant, indicating minimal leakage or degradation over this time interval.

Similarly, fluorescence emission spectra [Fig. 9(b)], in response to 750-nm excitation, and their corresponding values of σ [Fig. 9(c)] remained nearly unchanged for at least up to 7 days, further confirming the fluorescence stability of these RBC nano-ghosts. Interestingly, the peak emission intensity and the mean value of σ at day 0 for the dual-mode particles (Gd-BOPTA-BrCy112-nEGs), fabricated using 125 mM and 100  μM of Gd-BOPTA and BrCy112, respectively, were ∼2.6- and 2-fold higher, respectively, as compared with the values for the single-mode particles (BrCy112-nEGs) fabricated using 100  μM of BrCy112 [Figs. 7(c) and 7(e)]. This trend was maintained for the Gd-BOPTA-BrCy112-nEG particles at days 2 and 7 post-fabrication. Similarly, the mean value of ϕ for the dual-mode particles at day 0 [Fig. 9(d)] was about twofold higher than the value for single-mode particles [Fig. 7(f)] and remained higher up to 7 days post-fabrication. These enhanced fluorescence characteristics resemble a metal-induced-like enhancement in fluorescence when a fluorophore is within a near-field distance of a metal.^98^^,^^99^ Photoexcitation of the sub-wavelength Gd-BOPTA molecules may create and can enhance local electric field. In accordance with plasmon–fluorophore coupling,^100^ the presence of BrCy112 molecules in the vicinity of the intense electric field can enhance the intrinsic fluorescence emission of the dye.

MR images of Gd-BOPTA-BrCy112-nEG suspensions in 1X PBS obtained immediately after fabrication and at 2 and 7 days of storage at 4°C are shown in Fig. 9(e). There were minimal variations between the samples collected on days 0 and 7. Values of R1 remained nearly constant for up to 7 days following fabrication [Fig. 9(f)], indicating that there was minimal leakage (<1%) of Gd-BOPTA over this time interval.

### 3.4 Cytotoxicity

**Fig. 10:**
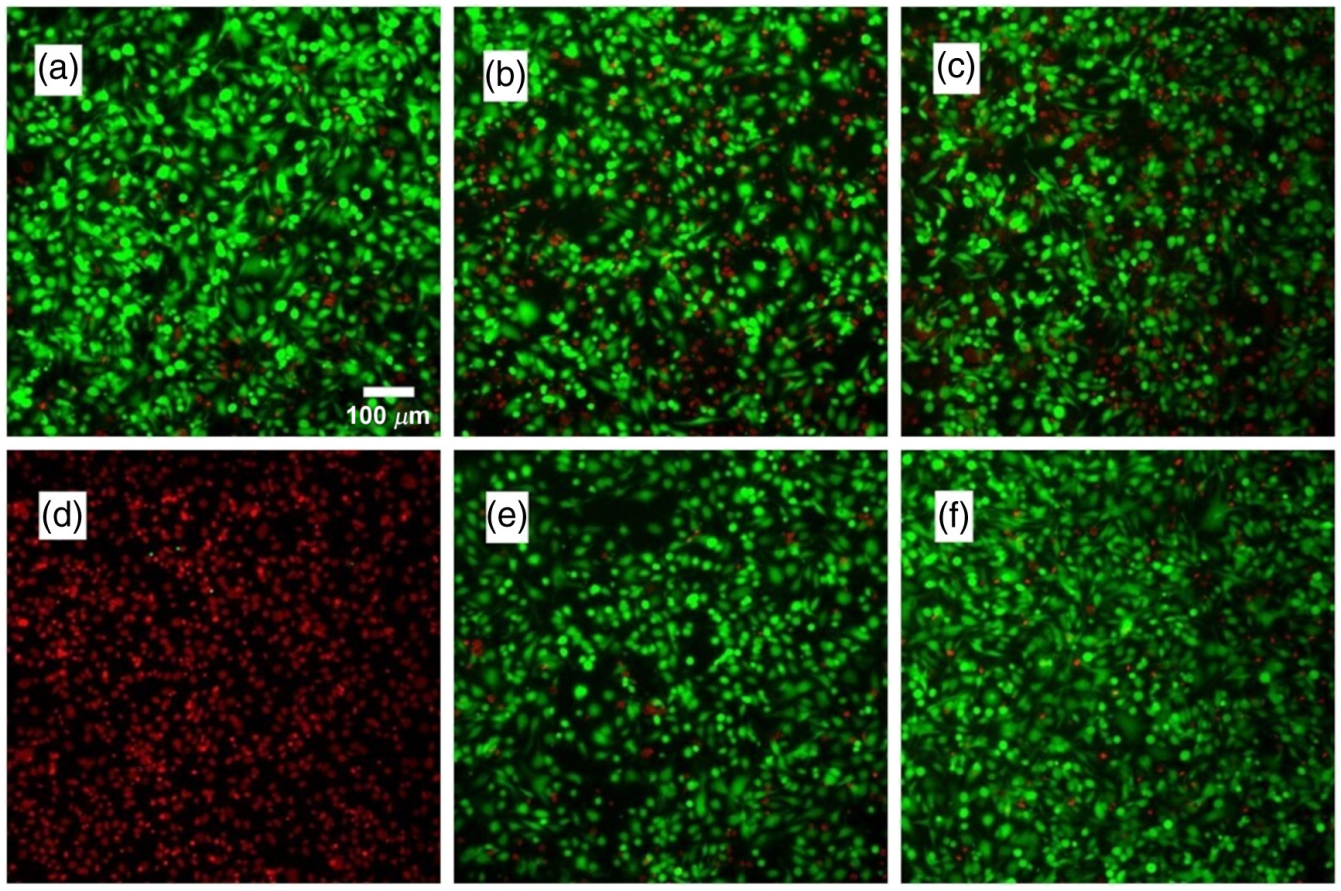
Fluorescence images of SKOV3 cells incubated with (a) culture medium (positive control) alone for 24 h, (b) culture medium containing free BrCy112 for 3 h, (c) culture medium containing free BrCy112 for 24 h, (d) methanol (negative control) for 24 h, (e) culture medium containing BrCy112-nEGs for 3 h, and (f) culture medium containing BrCy112-nEGs for 24 h.

Illustrative fluorescence images of SKOV3 cells incubated with culture medium alone (positive control), methanol (negative control), free BrCy112 (50  μM) added to the culture medium, or BrCy112-nEGs (fabricated using 100  μM BrCy112 in the loading buffer) added to the medium are shown in Fig. 10. Cells incubated with culture medium containing free BrCy112 or BrCy112-nEGs for 3 h [Figs. 10(b) and 10(e), respectively] or 24 h [Figs. 10(c) and 10(f), respectively] showed similar fluorescence emission characteristics as compared with the cells incubated with the culture medium without any other agents [Fig. 10(a)]. In contrast, cells incubated with methanol were dead as illustrated by the red fluorescence emission [Fig. 10(d)].

**Fig. 11:**
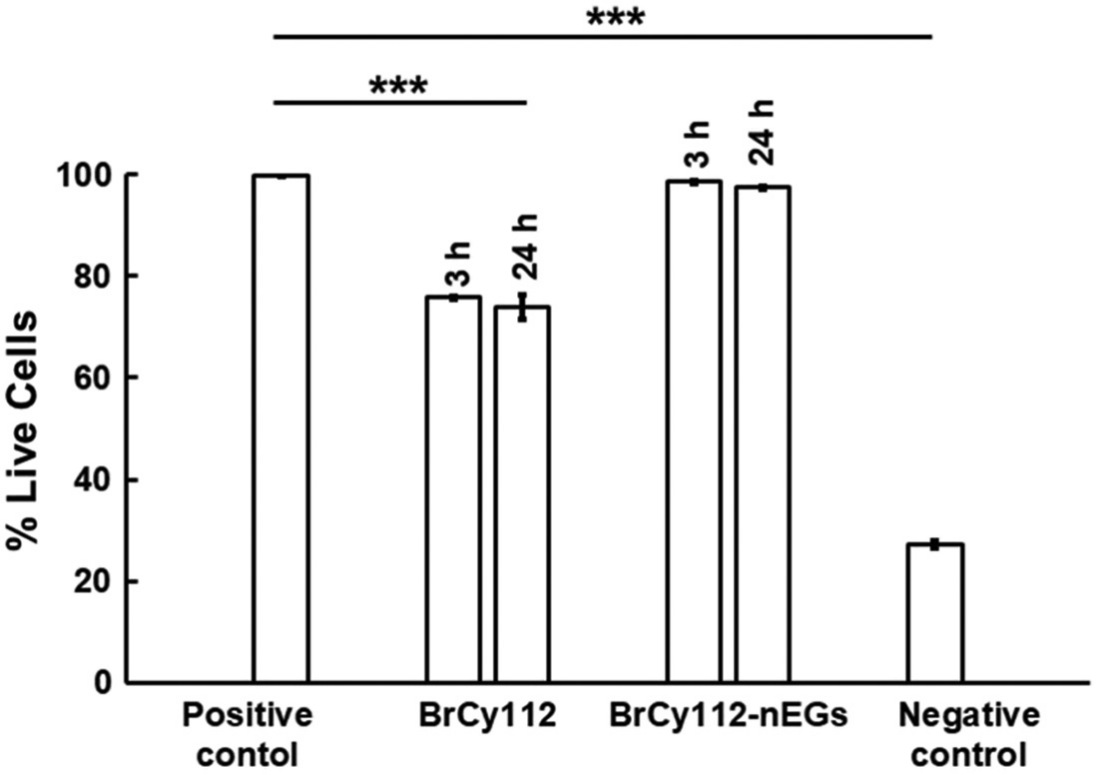
Quantitative cytotoxicity assessment as measured by the percentage of live SKOV3 cells following incubation with culture medium alone for 24 h (positive control), culture medium containing free BrCy112 for 3 and 24 h, culture medium containing BrCy112-nEGs for 3 and 24 h, and methanol for 24 h (negative control). Error bars represent standard deviations. Triple asterisks indicate statistically significant differences between the positive control and free BrCy112 and between positive and negative controls with p<0.001. There were no statistically significant differences in the mean fraction of live cells incubated with the cell culture medium without any other agents or the cell culture medium containing BrCy112-nEGs.

Quantitative cytotoxicity assessment based on the analysis of fluorescence images of SKOV cells is shown in Fig. 11. The mean values of the fraction of live cells following incubation with the cell culture medium containing free BrCy112 for 3 and 24 h were relatively high at ∼76% and 74%, respectively, but significantly lower than the value for cells incubated with the cell culture medium alone for 24 h (p<0.001). However, nearly 98% of the cells remained viable after incubation with the cell culture medium containing BrCy112-nEGs for 3 and 24 h, indicating that encapsulation of BrCy112 into the nano-sized EGs provides an effective method to shield the cells from the potential cytotoxic effects of the dye. Our future studies will include the use of appropriate animal models to evaluate the efficacy of the RBC nano-ghosts loaded with BrCy dyes (particularly BrCy106 and BrCy112) and Gd-BOPTA for dual optical and magnetic resonance imaging of tumors and to assess their safety *in vivo*.

## 4 Conclusion

We have engineered a nano-sized platform, derived from erythrocyte ghosts, with dual-NIR fluorescence and MR characteristics by co-encapsulation of a brominated carbocyanine dye and gadobenate dimeglumine, respectively. We find that the degree of bromination, structural symmetry, and acidic modifications have considerable influences on the fluorescence emission characteristics of RBC nano-ghosts. For example, nano-ghosts fabricated using 1000  μM of BrCy106, an unsymmetric dye with one dibromobenzene ring, and acidic modifications made to the indoline and indolenine groups demonstrate a nearly 54-fold increase in their relative fluorescence quantum yield as compared with those fabricated using the FDA-approved ICG. The dual-mode nano-ghosts containing BrCy112 and gadobenate dimeglumine show a nearly twofold increase in their relative fluorescence quantum yield as compared with the single-mode nano-constructs and retain their fluorescence and T1 relaxation rate characteristics for at least 7 days when stored at 4°C. Cytotoxicity is not observed upon incubation of SKOV3 cells with nano-ghosts containing BrCy112.

## Data Availability

Data are available from the authors upon request.

## References

[1] van Beurden F. et al., “Multi-wavelength fluorescence in image-guided surgery, clinical feasibility and future perspectives,” Mol. Imaging 19, 153601212096233 (2020). 10.1177/1536012120962333 PMC7607779 33125289

[r2] Teng C. W. et al., “Second window ICG predicts gross-total resection and progression-free survival during brain metastasis surgery,” J. Neurosurg. 135(4), 1026–1035 (2021). JONSAC 0022-3085 10.3171/2020.8.JNS201810 33652417 PMC10998541

[3] Zhang Y. Q. et al., “Novel self-assembled multifunctional nanoprobes for second-near-infrared-fluorescence-image-guided breast cancer surgery and enhanced radiotherapy efficacy,” Adv. Sci. 10(10), e2205294 (2023). 10.1002/advs.202205294 PMC10074043 36721054

[4] Frangioni J. V., “In vivo near-infrared fluorescence imaging,” Curr. Opin. Chem. Biol. 7(5), 626–634 (2003). COCBF4 1367-5931 10.1016/j.cbpa.2003.08.007 14580568

[r5] Hong G. S. Antaris A. L. Dai H. J., “Near-infrared fluorophores for biomedical imaging,” Nat. Biomed. Eng. 1(1), 0010 (2017). 10.1038/s41551-016-0010

[6] Hang Y. J. Boryczka J. Wu N. Q., “Visible-light and near-infrared fluorescence and surface-enhanced Raman scattering point-of-care sensing and bio-imaging: a review,” Chem. Soc. Rev. 51(1), 329–375 (2022). CSRVBR 0306-0012 10.1039/C9CS00621D 34897302 PMC9135580

[7] Desmettre T. Devoisselle J. M. Mordon S., “Fluorescence properties and metabolic features of indocyanine green (ICG) as related to angiography,” Surv. Ophthalmol. 45(1), 15–27 (2000). SUOPAD 0039-6257 10.1016/S0039-6257(00)00123-5 10946079

[8] Vos J. J. et al., “Green light for liver function monitoring using indocyanine green? An overview of current clinical applications,” Anaesthesia 69(12), 1364–1376 (2014). 10.1111/anae.12755 24894115

[9] van der Vorst J. R. et al., “Near-infrared fluorescence-guided resection of colorectal liver metastases,” Cancer 119(18), 3411–3418 (2013). CANCAR 0008-543X 10.1002/cncr.28203 23794086 PMC3775857

[r10] Ushimaru Y. et al., “The feasibility and safety of preoperative fluorescence marking with indocyanine green (ICG) in laparoscopic gastrectomy for gastric cancer,” J. Gastrointest. Surg. 23(3), 468–476 (2019). 10.1007/s11605-018-3900-0 30084063

[r11] Peltrini R. et al., “Intraoperative use of indocyanine green fluorescence imaging in rectal cancer surgery: the state of the art,” World J. Gastroenterol. 27(38), 6374–6386 (2021). 10.3748/wjg.v27.i38.6374 34720528 PMC8517789

[r12] Tokumaru S. et al., “Intraoperative visualization of morphological patterns of the thoracic duct by subcutaneous inguinal injection of indocyanine green in esophagectomy for esophageal cancer,” Ann. Gastroenterol. Surg. 6(6), 873–879 (2022). 10.1002/ags3.12594 36338584 PMC9628221

[13] Gong M. F. et al., “Intraoperative evaluation of soft tissue sarcoma surgical margins with indocyanine green fluorescence imaging,” Cancers 15(3), 582 (2023). 10.3390/cancers15030582 36765538 PMC9913765

[14] Levitz A. et al., “Synthesis and effect of heterocycle modification on the spectroscopic properties of a series of unsymmetrical trimethine cyanine dyes,” Dyes Pigments 105, 238–249 (2014). 10.1016/j.dyepig.2014.02.009

[15] Fox I. J. Wood E. H., “Applications of dilution curves recorded from the right side of the heart or venous circulation with the aid of a new indicator dye,” in Proc. Staff Meet. Mayo Clin., Vol. 32, pp. 541–550 (1957). 13465840

[16] Philip R. et al., “Absorption and fluorescence spectroscopic investigation of indocyanine green,” J. Photochem. Photobiol. A 96(1–3), 137–148 (1996). JPPCEJ 1010-6030 10.1016/1010-6030(95)04292-X

[17] Meijer D. K. Weert B. Vermeer G. A., “Pharmacokinetics of biliary excretion in man. VI. Indocyanine green,” Eur. J. Clin. Pharmacol. 35(3), 295–303 (1988). EJCPAS 0031-6970 10.1007/BF00558268 3181282

[18] Jia W. et al., “Intravital vascular phototheranostics and real-time circulation dynamics of micro- and nanosized erythrocyte-derived carriers,” ACS Appl. Mater. Interfaces 12(1), 275–287 (2020). AAMICK 1944-8244 10.1021/acsami.9b18624 31820920 PMC7028219

[19] Cherrick G. R. et al., “Indocyanine green: observations on its physical properties, plasma decay, and hepatic extraction,” J. Clin. Invest. 39(4), 592–600 (1960). JCINAO 0021-9738 10.1172/JCI104072 13809697 PMC293343

[r20] Yoneya S. et al., “Binding properties of indocyanine green in human blood,” Invest. Ophthalmol. Vis. Sci. 39(7), 1286–1290 (1998). IOVSDA 0146-0404 9620093

[21] Faybik P. Hetz H., “Plasma disappearance rate of indocyanine green in liver dysfunction,” Transplant. Proc. 38(3), 801–802 (2006). TRPPA8 0041-1345 10.1016/j.transproceed.2006.01.049 16647475

[22] Nolting D. D. Gore J. C. Pham W., “Near-infrared dyes: probe development and applications in optical molecular imaging,” Curr. Org. Synth. 8(4), 521–534 (2011). 10.2174/157017911796117223 21822405 PMC3150548

[r23] Yuan L. et al., “A unique class of near-infrared functional fluorescent dyes with carboxylic-acid-modulated fluorescence ON/OFF switching: rational design, synthesis, optical properties, theoretical calculations, and applications for fluorescence imaging in living animals,” J. Am. Chem. Soc. 134(2), 1200–1211 (2012). JACSAT 0002-7863 10.1021/ja209292b 22176300

[r24] Liu J. et al., “Sulfone-rhodamines: a new class of near-infrared fluorescent dyes for bioimaging,” ACS Appl. Mater. Interfaces 8(35), 22953–22962 (2016). AAMICK 1944-8244 10.1021/acsami.6b08338 27548811

[r25] Sun Y. et al., “Novel benzo-bis(1,2,5-thiadiazole) fluorophores for in vivo NIR-II imaging of cancer,” Chem. Sci. 7(9), 6203–6207 (2016).1478-6524 10.1039/C6SC01561A 30034761 PMC6024204

[r26] Ding F. et al., “Recent advances in near-infrared II fluorophores for multifunctional biomedical imaging,” Chem. Sci. 9(19), 4370–4380 (2018).1478-6524 10.1039/C8SC01153B 29896378 PMC5961444

[r27] Jiao M. X. et al., “Semiconductor nanocrystals emitting in the second near-infrared window: optical properties and application in biomedical imaging,” Adv. Opt. Mater. 10(14), 2200226 (2022).2195-1071 10.1002/adom.202200226

[28] Medeiros N. G. et al., “Near-infrared fluorophores based on heptamethine cyanine dyes: from their synthesis and photophysical properties to recent optical sensing and bioimaging applications,” Asian J. Org. Chem. 11(6), 118–149 (2022). 10.1002/ajoc.202200095

[29] Liu H. et al., “Halogenated cyanine dyes for synergistic photodynamic and photothermal therapy,” Dyes Pigments 190, 109327 (2021). 10.1016/j.dyepig.2021.109327

[r30] Jones G. W. Tatarets A. L. Patsenker L. D., “Halogenated compounds for photodynamic therapy,” US 8,962,797 B2 (2015).

[r31] Kobzev D. et al., “Antibody-guided iodinated cyanine for near-IR photoimmunotherapy,” Dyes Pigments 212, 111101 (2023). 10.1016/j.dyepig.2023.111101

[32] Atchison J. et al., “Iodinated cyanine dyes: a new class of sensitisers for use in NIR activated photodynamic therapy (PDT),” Chem. Commun. 53(12), 2009–2012 (2017). 10.1039/C6CC09624G 28124050

[33] Guerrero Y. et al., “Optical characteristics and tumor imaging capabilities of near infrared dyes in free and nano-encapsulated formulations comprised of viral capsids,” ACS Appl. Mater. Interfaces 9(23), 19601–19611 (2017). AAMICK 1944-8244 10.1021/acsami.7b03373 28524652

[34] Vankayala R. et al., “Virus-mimicking nanoparticles for targeted near infrared fluorescence imaging of intraperitoneal ovarian tumors in mice,” Ann. Biomed. Eng. 49(2), 548–559 (2021). ABMECF 0090-6964 10.1007/s10439-020-02589-8 32761557

[35] Kirchherr A. K. Briel A. Mader K., “Stabilization of indocyanine green by encapsulation within micellar systems,” Mol. Pharmaceut. 6(2), 480–491 (2009). MPOHBP 1543-8384 10.1021/mp8001649 19228053

[r36] Saxena V. Sadoqi M. Shao J., “Enhanced photo-stability, thermal-stability and aqueous-stability of indocyanine green in polymeric nanoparticulate systems,” J. Photochem. Photobiol. B 74(1), 29–38 (2004). JPPBEG 1011-1344 10.1016/j.jphotobiol.2004.01.002 15043844

[r37] Yu J. et al., “Synthesis of near-infrared-absorbing nanoparticle-assembled capsules,” Chem. Mater. 19(6), 1277–1284 (2007). CMATEX 0897-4756 10.1021/cm062080x

[r38] Yaseen M. A. et al., “In-vivo fluorescence imaging of mammalian organs using charge-assembled mesocapsule constructs containing indocyanine green,” Opt. Express 16(25), 20577–20587 (2008). OPEXFF 1094-4087 10.1364/OE.16.020577 19065196

[r39] Bahmani B. et al., “Effect of polyethylene glycol coatings on uptake of indocyanine green loaded nanocapsules by human spleen macrophages in vitro,” J. Biomed. Opt. 16(5), 051303 (2011). JBOPFO 1083-3668 10.1117/1.3574761 21639563

[r40] Bahmani B. et al., “Effects of nanoencapsulation and PEGylation on biodistribution of indocyanine green in healthy mice: quantitative fluorescence imaging and analysis of organs,” Int. J. Nanomed. 8, 1609–1620 (2013). 10.2147/IJN.S42511 PMC3635661 23637530

[r41] Gupta S. et al., “Virus-mimicking nano-constructs as a contrast agent for near infrared photoacoustic imaging,” Nanoscale 5(5), 1772–1776 (2013). NANOHL 2040-3364 10.1039/c3nr34124k 23334567 PMC3626106

[r42] Jian W. H. et al., “Indocyanine green-encapsulated hybrid polymeric nanomicelles for photothermal cancer therapy,” Langmuir 31(22), 6202–6210 (2015). LANGD5 0743-7463 10.1021/acs.langmuir.5b00963 25985856

[r43] Lee Y. H. Chang D. S., “Fabrication, characterization, and biological evaluation of anti-HER2 indocyanine green-doxorubicin-encapsulated PEG-b-PLGA copolymeric nanoparticles for targeted photochemotherapy of breast cancer cells,” Sci. Rep. 7, 46688 (2017). SRCEC3 2045-2322 10.1038/srep46688 28429764 PMC5399361

[r44] Okamoto Y. et al., “Inhibitory effects and gene expression analysis of chemotherapeutic photodynamic therapy by using a liposomally formulated indocyanine green derivative,” Photodiagn. Photodyn. Ther. 39, 102961 (2022). 10.1016/j.pdpdt.2022.102961 35700912

[45] Liu Y. et al., “Liposome-based multifunctional nanoplatform as effective therapeutics for the treatment of retinoblastoma,” Acta Pharm. Sin. B 12(6), 2731–2739 (2022). 10.1016/j.apsb.2021.10.009 35755292 PMC9214327

[46] Bahmani B. Bacon D. Anvari B., “Erythrocyte-derived photo-theranostic agents: hybrid nano-vesicles containing indocyanine green for near infrared imaging and therapeutic applications,” Sci. Rep. 3, 2180 (2013). SRCEC3 2045-2322 10.1038/srep02180 23846447 PMC3709166

[47] Burns J. M. et al., “Erythrocyte-derived theranostic nanoplatforms for near infrared fluorescence imaging and photodestruction of tumors,” ACS Appl. Mater. Interfaces 10(33), 27621–27630 (2018). AAMICK 1944-8244 10.1021/acsami.8b08005 30036031 PMC6526021

[r48] Burns J. M. et al., “Near infrared fluorescence imaging of intraperitoneal ovarian tumors in mice using erythrocyte-derived optical nanoparticles and spatially-modulated illumination,” Cancers 13(11), 2544 (2021). 10.3390/cancers13112544 34067308 PMC8196853

[49] Mac J. T. et al., “Erythrocyte-derived nanoparticles with folate functionalization for near infrared pulsed laser-mediated photo-chemotherapy of tumors,” Int. J. Mol. Sci. 23(18), 10295 (2022).1422-0067 10.3390/ijms231810295 36142205 PMC9499474

[50] Guido C. et al., “Erythrocytes and nanoparticles: new therapeutic systems,” Appl. Sci. 11(5), 2173 (2021). 10.3390/app11052173

[r51] Luk B. T. et al., “Safe and immunocompatible nanocarriers cloaked in RBC membranes for drug delivery to treat solid tumors,” Theranostics 6(7), 1004–1011 (2016). 10.7150/thno.14471 27217833 PMC4876624

[r52] Huang S. N. et al., “Red blood cell membrane-coated functionalized Au nanocage as a biomimetic platform for improved microRNA delivery in hepatocellular carcinoma,” Int. J. Pharmaceut. 642, 123044 (2023). 10.1016/j.ijpharm.2023.123044 37178790

[53] Zhang L. et al., “Erythrocyte membrane cloaked metal-organic framework nanoparticle as biomimetic nanoreactor for starvation-activated colon cancer therapy,” ACS Nano 12(10), 10201–10211 (2018). ANCAC3 1936-0851 10.1021/acsnano.8b05200 30265804

[54] Bentley A. A. Adams J. C., “The evolution of thrombospondins and their ligand-binding activities,” Mol. Biol. Evol. 27(9), 2187–2197 (2010). MBEVEO 0737-4038 10.1093/molbev/msq107 20427418 PMC3107593

[r55] Yoo J. W. et al., “Bio-inspired, bioengineered and biomimetic drug delivery carriers,” Nat. Rev. Drug Discov. 10(7), 521–535 (2011). NRDDAG 1474-1776 10.1038/nrd3499 21720407

[r56] Oldenborg P. A., “CD47: a cell surface glycoprotein which regulates multiple functions of hematopoietic cells in health and disease,” ISRN Hematol. 2013, 614619 (2013). 10.1155/2013/614619 23401787 PMC3564380

[57] Rodriguez P. L. et al., “Minimal “self” peptides that inhibit phagocytic clearance and enhance delivery of nanoparticles,” Science 339(6122), 971–975 (2013). SCIEAS 0036-8075 10.1126/science.1229568 23430657 PMC3966479

[58] Lee C. H. et al., “Proteomes of micro- and nanosized carriers engineered from red blood cells,” J. Proteome Res. 22(3), 896–907 (2023). 10.1021/acs.jproteome.2c00695 36792548 PMC10756254

[59] Song W. T. et al., “Comprehensive studies of pharmacokinetics and biodistribution of indocyanine green and liposomal indocyanine green by multispectral optoacoustic tomography,” RSC Adv. 5(5), 3807–3813 (2015). 10.1039/C4RA09735A

[60] Vankayala R. et al., “Biodistribution and toxicological evaluation of micron- and nano-sized erythrocyte-derived optical particles in healthy Swiss Webster mice,” Biomater. Sci. 7(5), 2123–2133 (2019). 10.1039/C8BM01448E 30869663 PMC9844153

[61] Hanley T. M. et al., “Acute immune response of micro- and nanosized erythrocyte-derived optical particles in healthy mice,” Mol. Pharm. 17(10), 3900–3914 (2020). 10.1021/acs.molpharmaceut.0c00641 32820927 PMC9844151

[62] Poss M. et al., “Multimodal ${\left[\mathrm{GdO}\right]}^{+}{\left[\mathrm{ICG}\right]}^{-}$ nanoparticles for optical, photoacoustic, and magnetic resonance imaging,” Chem. Mater. 29(8), 3547–3554 (2017). CMATEX 0897-4756 10.1021/acs.chemmater.6b05406

[63] Sharma P. et al., “Gadolinium-doped silica nanoparticles encapsulating indocyanine green for near infrared and magnetic resonance imaging,” Small 8(18), 2856–2868 (2012). SMALBC 1613-6810 10.1002/smll.201200258 22744832

[r64] Cao M. J. et al., “Gadolinium(III)-chelated silica nanospheres integrating chemotherapy and photothermal therapy for cancer treatment and magnetic resonance imaging,” ACS Appl. Mater. Interfaces 7(45), 25014–25023 (2015). AAMICK 1944-8244 10.1021/acsami.5b06938 26418578

[65] Ravoori M. K. et al., “Multimodal magnetic resonance and near-infrared-fluorescent imaging of intraperitoneal ovarian cancer using a dual-mode-dual-gadolinium liposomal contrast agent,” Sci. Rep. 6, 38991 (2016). SRCEC3 2045-2322 10.1038/srep38991 28004770 PMC5177955

[66] Chanana M. et al., “Fabrication of colloidal stable, thermosensitive, and biocompatible magnetite nanoparticles and study of their reversible agglomeration in aqueous milieu,” Chem. Mater. 21(9), 1906–1914 (2009). CMATEX 0897-4756 10.1021/cm900126r

[67] Chang M. et al., “Homologous RBC-derived vesicles as ultrasmall carriers of iron oxide for magnetic resonance imaging of stem cells,” Nanotechnology 21(23), 235103 (2010). NNOTER 0957-4484 10.1088/0957-4484/21/23/235103 20479509

[68] Kirchin M. A. Pirovano G. P. Spinazzi A., “Gadobenate dimeglumine (Gd-BOPTA). An overview,” Invest. Radiol. 33(11), 798–809 (1998). INVRAV 0020-9996 10.1097/00004424-199811000-00003 9818314

[69] Runge V. M. et al., “A clinical comparison of the safety and efficacy of MultiHance (gadobenate dimeglumine) and Omniscan (gadodiamide) in magnetic resonance imaging in patients with central nervous system pathology,” Invest. Radiol. 36(2), 65–71 (2001). INVRAV 0020-9996 10.1097/00004424-200102000-00001 11224753

[r70] Essig M., “Gadobenate dimeglumine (MultiHance) in MR imaging of the CNS: studies to assess the benefits of a high relaxivity contrast agent,” Acad. Radiol. 12 Suppl 1, S23–S27 (2005). 10.1016/j.acra.2005.02.020 16106542

[r71] Thurnher S. et al., “Diagnostic performance of gadobenate dimeglumine enhanced MR angiography of the iliofemoral and calf arteries: a large-scale multicenter trial,” AJR Am. J. Roentgenol. 189(5), 1223–1237 (2007). 10.2214/AJR.07.2218 17954665

[r72] Iezzi R. et al., “Contrast-enhanced MRA of the renal and aorto-iliac-femoral arteries: comparison of gadobenate dimeglumine and gadofosveset trisodium,” Eur. J. Radiol. 77(2), 358–368 (2011). EJRADR 0720-048X 10.1016/j.ejrad.2009.07.020 19679417

[73] Heshmatzadeh Behzadi A. McDonald J., “Gadolinium-based contrast agents for imaging of the central nervous system: a multicenter European prospective study,” Medicine 101(34), e30163 (2022). MEDIAV 0025-7974 10.1097/MD.0000000000030163 36042629 PMC9410688

[74] Auersperg N. et al., “Ovarian surface epithelium: biology, endocrinology, and pathology,” Endocr. Rev. 22(2), 255–288 (2001). ERVIDP 0163-769X 10.1210/edrv.22.2.0422 11294827

[75] Cosco E. D. Lim I. Sletten E. M., “Photophysical properties of indocyanine green in the shortwave infrared region,” ChemPhotoChem 5(8), 727–734 (2021). 10.1002/cptc.202100045 34504949 PMC8423351

[76] Thavornpradit S. et al., “QuatCy: a heptamethine cyanine modification with improved characteristics,” Theranostics 9(10), 2856–2867 (2019). 10.7150/thno.33595 31244928 PMC6568187

[77] Valdesaguilera O. Neckers D. C., “Aggregation phenomena in xanthene dyes,” Accounts Chem. Res. 22(5), 171–177 (1989). 10.1021/ar00161a002

[78] Ogawa M. et al., “H-type dimer formation of fluorophores: a mechanism for activatable, in vivo optical molecular imaging,” ACS Chem. Biol. 4(7), 535–546 (2009). 10.1021/cb900089j 19480464 PMC2743556

[79] Herz A. H., “Aggregation of sensitizing dyes in solution and their adsorption onto silver halides,” Adv. Colloid Interface Sci. 8(4), 237–298 (1977). ACISB9 0001-8686 10.1016/0001-8686(77)80011-0

[80] Kasha M., “Energy transfer mechanisms and the molecular exciton model for molecular aggregates,” Radiat. Res. 20, 55–70 (1963). RAREAE 0033-7587 10.2307/3571331 14061481

[81] Afanasenko A. M. et al., “Intermolecular interactions-photophysical properties relationships in phenanthrene-9,10-dicarbonitrile assemblies,” J. Mol. Struct. 1199, 126789 (2020). JMOSB4 0022-2860 10.1016/j.molstruc.2019.07.036

[82] Zhai L. et al., “Bromine-functionalized covalent organic frameworks for efficient triboelectric nanogenerator,” Chemistry 26(26), 5784–5788 (2020). CHRYAQ 0009-305X 10.1002/chem.202000722 32073179

[83] Biffinger J. C. Kim H. W. DiMagno S. G., “The polar hydrophobicity of fluorinated compounds,” ChemBioChem 5(5), 622–627 (2004). CBCHFX 1439-4227 10.1002/cbic.200300910 15122633

[84] Bifari E. N. et al., “Synthesis, photophysical, electrochemical and computational investigation of dimethine and trimethine cyanine-based dyes,” J. Photochem. Photobiol. A 433, 114189 (2022). JPPCEJ 1010-6030 10.1016/j.jphotochem.2022.114189

[85] Cheng M. et al., “Dye-sensitized solar cells based on a donor-acceptor system with a pyridine cation as an electron-withdrawing anchoring group,” Chem.-Eur. J. 18(50), 16196–16202 (2012). 10.1002/chem.201200826 23081757

[86] Bricks J. L. et al., “Fluorescent J-aggregates of cyanine dyes: basic research and applications review,” Methods Appl. Fluoresc. 6(1), 012001 (2018). 10.1088/2050-6120/aa8d0d 28914610

[r87] Bertocchi F. et al., “Aggregates of cyanine dyes: when molecular vibrations and electrostatic screening make the difference,” J. Phys. Chem. C Nanomater. Interfaces 127(21), 10185–10196 (2023). 10.1021/acs.jpcc.3c01253 37284292 PMC10240496

[88] Matsui M. et al., “Fluorescence properties of indolenium carbocyanine dyes in solid state,” Tetrahedron 71(21), 3528–3534 (2015). TETRAB 0040-4020 10.1016/j.tet.2015.03.027

[89] Xiang J. et al., “Effects of NaCl on the J-aggregation of two thiacarbocyanine dyes in aqueous solutions,” J. Colloid Interface Sci. 258(1), 198–205 (2003). JCISA5 0021-9797 10.1016/S0021-9797(02)00187-X 12600788

[90] Chibisov A. K. Gorner H. Slavnova T. D., “Kinetics of salt-induced J-aggregation of an anionic thiacarbocyanine dye in aqueous solution,” Chem. Phys. Lett. 390(1–3), 240–245 (2004). CHPLBC 0009-2614 10.1016/j.cplett.2004.03.131

[91] Xu M. et al., “Synthesis and study on aggregation behaviours in liquid phase of three prepared cyanine dyes,” Luminescence 37(10), 1733–1740 (2022). 10.1002/bio.4349 35894773

[92] Jung B. S. Vullev V. I. Anvari B., “Revisiting indocyanine green: effects of serum and physiological temperature on absorption and fluorescence characteristics,” IEEE J. Sel. Top. Quantum Electron. 20(2), 149–157 (2014). IJSQEN 1077-260X 10.1109/JSTQE.2013.2278674

[93] Shao Y. et al., “How does the interplay between bromine substitution at bay area and bulky substituents at imide position influence the photophysical properties of perylene diimides?” RSC Adv. 7(26), 16155–16162 (2017). 10.1039/C7RA00779E

[94] Whitaker J. R. Granum P. E., “An absolute method for protein determination based on difference in absorbance at 235 and 280 nm,” Anal. Biochem. 109(1), 156–159 (1980). ANBCA2 0003-2697 10.1016/0003-2697(80)90024-X 7469012

[r95] Stoscheck C. M., “Quantitation of protein,” Method Enzymol. 182, 50–68 (1990). 10.1016/0076-6879(90)82008-P 2314256

[96] Noble J. E., “Quantification of protein concentration using UV absorbance and Coomassie dyes,” Methods Enzymol. 536, 17–26 (2014). MENZAU 0076-6879 10.1016/B978-0-12-420070-8.00002-7 24423263

[97] Eylar E. H. et al., “The contribution of sialic acid to the surface charge of the erythrocyte,” J. Biol. Chem. 237, 1992–2000 (1962). JBCHA3 0021-9258 10.1016/S0021-9258(19)73972-6 13891108

[98] Ray K. Lakowicz J. R., “Metal-enhanced fluorescence lifetime imaging and spectroscopy on a modified SERS substrate,” J. Phys. Chem. C Nanomater. Interfaces 117(30), 15790–15797 (2013). 10.1021/jp404590j PMC3886561 24416457

[99] Ray K. et al., “Several hundred-fold enhanced fluorescence from single fluorophores assembled on silver nanoparticle-dielectric-metal substrate,” Chem. Commun. 51(81), 15023–15026 (2015). 10.1039/C5CC03581C PMC4893342 26312260

[100] Lakowicz J. R. et al., “Plasmon-controlled fluorescence: a new paradigm in fluorescence spectroscopy,” Analyst 133(10), 1308–1346 (2008). ANLYAG 0365-4885 10.1039/b802918k 18810279 PMC2710039

